# Engrafted nitrergic neurons derived from hPSCs improve gut dysmotility in mice

**DOI:** 10.1038/s41586-025-09208-3

**Published:** 2025-06-25

**Authors:** Homa Majd, Ryan M. Samuel, Andrius Cesiulis, Jonathan T. Ramirez, Ali Kalantari, Kevin Barber, Sina Farahvashi, Zaniar Ghazizadeh, Alireza Majd, Angeline K. Chemel, Mikayla N. Richter, Subhamoy Das, Jacqueline L. Bendrick, Matthew G. Keefe, Jeffrey Wang, Rahul K. Shiv, Samyukta Bhat, Matvei Khoroshkin, Johnny Yu, Tomasz J. Nowakowski, Kwun Wah Wen, Hani Goodarzi, Nikhil Thapar, Julia A. Kaltschmidt, Conor J. McCann, Faranak Fattahi

**Affiliations:** 1 Department of Cellular and Molecular Pharmacology, University of California, San Francisco, San Francisco, CA USA; 2 Eli and Edythe Broad Center of Regeneration Medicine and Stem Cell Research, University of California, San Francisco, San Francisco, CA USA; 3 Division of Cardiovascular Medicine, School of Medicine, Stanford University, Stanford, CA USA; 4 Department of Neurosurgery, School of Medicine, Stanford University, Stanford, CA USA; 5 Stanford Neurosciences Interdepartmental Program, Stanford University, Stanford, CA USA; 6 Wu Tsai Neurosciences Institute, Stanford University, Stanford, CA USA; 7 Department of Anatomy, University of California, San Francisco, San Francisco, CA USA; 8 Department of Psychiatry and Behavioral Sciences, University of California, San Francisco, San Francisco, CA USA; 9 Department of Biochemistry and Biophysics, University of California, San Francisco, San Francisco, CA USA; 10 Department of Electrical Engineering, Stanford University, Stanford, CA USA; 11 Department of Neurological Surgery, University of California, San Francisco, San Francisco, CA USA; 12 Department of Pathology, University of California, San Francisco, San Francisco, CA USA; 13 Arc Institute, Palo Alto, CA USA; 14 Stem Cells and Regenerative Medicine, UCL Great Ormond Street Institute of Child Health, London, UK; 15 Gastroenterology, Hepatology and Liver Transplant, Queensland Children’s Hospital, Brisbane, Queensland Australia; 16 School of Medicine, University of Queensland, Brisbane, Queensland Australia; 17 Centre for Child Nutrition Research, Queensland University of Technology, Brisbane, Queensland Australia; 18 NIHR Great Ormond Street Hospital BRC, London, UK

**Keywords:** Stem-cell differentiation, Regeneration and repair in the nervous system, Differentiation, Regeneration

## Abstract

Gastrointestinal (GI) motility disorders represent a major medical challenge, with few effective therapies available. These disorders often result from dysfunction of inhibitory nitric oxide (NO)-producing motor neurons in the enteric nervous system, which are essential for regulating gut motility. Loss or dysfunction of NO neurons is linked to severe conditions, including achalasia, gastroparesis, intestinal pseudo-obstruction and chronic constipation^1,2^. Here we introduce a platform based on human pluripotent stem cells (hPSCs) for therapeutic development targeting GI motility disorders. Using an unbiased screen, we identified drug candidates that modulate NO neuron activity and enhance motility in mouse colonic tissue ex vivo. We established a high-throughput strategy to define developmental programs driving the specification of NO neurons and found that inhibition of platelet-derived growth factor receptors (PDGFRs) promotes their differentiation from precursors of the enteric nervous system. Transplantation of these neurons into NO-neuron-deficient mice led to robust engraftment and improved GI motility, offering a promising cell-based therapy for neurodegenerative GI disorders. These studies provide a new framework for understanding and treating enteric neuropathies.

## Abstract (web summary)

A study presents a strategy based on human pluripotent stem cells for treating neurodegenerative disorders of the gastrointestinal tract.

## Main

GI motility disorders, including enteric neuropathies and disorders of gut–brain interaction, are highly prevalent and clinically challenging, affecting millions worldwide^3^. These conditions arise from degeneration or dysfunction of the enteric nervous system (ENS)^4–10^, which controls gut motility independently of the central nervous system (CNS)^11^. Limited understanding of ENS development and function contributes to the lack of effective therapies.

Enteric NO-producing neurons (nitrergic or NO neurons), which promote motility by releasing nitric oxide to relax smooth muscle, are particularly implicated in GI dysmotility. Their selective dysfunction or loss is associated with disorders such as achalasia, hypertrophic pyloric stenosis, and idiopathic and diabetic gastroparesis^1,2^. However, studying human NO neurons has been difficult owing to limited tissue access, low yield and challenges in maintaining functional neurons in vitro.

Overcoming these barriers could enable large-scale characterization, drug screening and cell therapy development. Here we present a system based on hPSCs to generate and study enteric NO neurons. Our scalable two-dimensional (2D) ENS cultures and 3D ganglioids provide platforms for molecular and functional analysis, pharmacological testing and regenerative applications.

Using these models, we identified pathways regulating NO neuron specification through a high-throughput small-molecule screen and established a robust differentiation protocol for generating enriched NO neuron populations. We further demonstrated their functional potential by modulating motility ex vivo and achieving successful engraftment in the colon of a mouse model for GI motility disorder. These findings introduce a new strategy for regenerative therapy and offer a model for future studies of human enteric NO neurons in vivo.

## Enteric ganglioids model the human ENS

**Extended Data Fig. 1:**
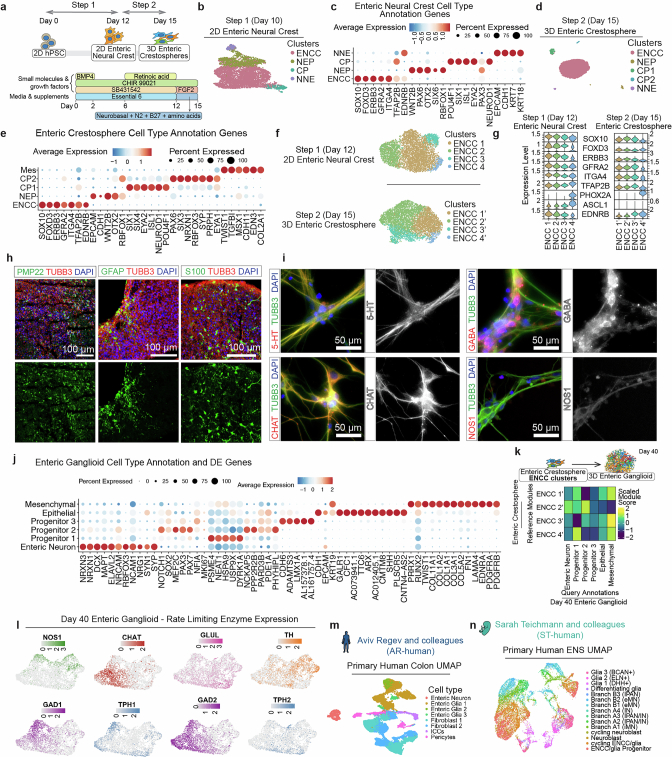
Cell-type specific molecular characterization of different developmental stages of hPSC-deried ENS models. **a**) Protocol schematic for in vitro differentiation and maturation of hPSCs into enteric neural crest and enteric crestospheres. **b**) scRNA-seq UMAP of clusters present in enteric neural crest cells (day 10) of the differentiation cultures depicted in (**a**). **c**) Dot plot of the scaled average expression of cluster annotation genes for enteric neural crest clusters. All data are derived from scRNA-seq analysis. **d**) scRNA-seq UMAP of clusters present in enteric crestosphere cells (day 15) of the differentiation cultures depicted in (**a**). **e**) Dot plot of the scaled average expression of cluster annotation genes for enteric crestosphere clusters. All data are derived from scRNA-seq analysis. **f**) UMAP of enteric neural crest (day 10, top) and enteric crestosphere (day 15, bottom) subtypes in differentiation cultures. **g**) Violin plot stack showing the expression of canonical enteric neural crest markers in enteric neural crest (top) and enteric crestosphere (bottom) subtypes. **h**) Immunofluorescence analysis of glial lineage markers PMP22, GFAP and S100 in day 80 enteric ganglioids. **i**) Immunofluorescence analysis for expression of ENS cell-type markers (serotonin (5-HT), CHAT, GABA and NOS1) in day 40 2D ENS cultures. **j**) Dot plot of the scaled average expression of cluster annotation genes for day 40 enteric ganglioid. **k**) Heatmap of the average module scores of enteric crestosphere (day 15) cluster transcriptional signatures in day 40 enteric ganglioid clusters. **l**) Feature plots showing the expression of rate limiting enzymes in neurotransmitter synthesis pathways by day 40 enteric ganglioid neurons. **m**,**n**) UMAPs of the primary human datasets AR-human (**m**) and ST-human (**n**) used for comparisons with hPSC-derived ENS cultures.

The ENS is predominantly derived from the vagal neural crest (NC)^12–15^. We have previously established hPSC differentiation methods to derive vagal enteric neural crest cells (ENCCs) under highly defined conditions^16,17^ (Extended Data Fig. 1a). This protocol involves two steps that follow embryonic NC development. In step 1, we induce ENCCs by activating bone morphogenic protein (BMP) and WNT signalling in combination with retinoic acid treatment. Retinoic acid caudalizes the differentiating NC, specifying a vagal NC identity. In step 2, we generate enteric crestospheres in the presence of WNT and fibroblast growth factor (FGF) signalling^16,17^.

To further characterize the differentiation of ENCCs and enteric crestospheres, we performed single-cell RNA sequencing. During the ENCC stage, four transcriptionally distinct clusters are present: ENCC (SOX10^+^FOXD3^+^), neuro-epithelial progenitor (WNT2B^+^PAX6^+^), cranial placode (SIX1^+^EYA2^+^) and non-neural ectoderm (EPCAM^+^CDH1^+^; Extended Data Fig. 1b,c). In the next step, suspension culture of ENCCs serves as a purification strategy that leads to enteric crestospheres consisting primarily of ENCCs with a small population of neuro-epithelial progenitors, two cranial placode clusters (CP1 and CP2) and a mesenchymal (TWIST1^+^MSX1^+^) cluster (Extended Data Fig. 1d,e). Further subclustering of the ENCC populations identified four clusters (ENCC 1–4 for the enteric NC stage and ENCC 1′–4′ for the enteric crestospheres) that showed differential expression of some of the canonical ENCC markers (Extended Data Fig. 1f,g).

hPSC-derived ENCCs have been shown to recapitulate developmental migration defects in Hirschsprung disease^16,17^. However, their capacity to form the diverse array of neuronal and glial cell types that make up the ENS remains unexplored. The next pivotal step is to continue the differentiation of ENCCs as they progress towards the ENS, providing a model system to study a wide range of ENS disorders, beyond developmental defects.

**Fig. 1:**
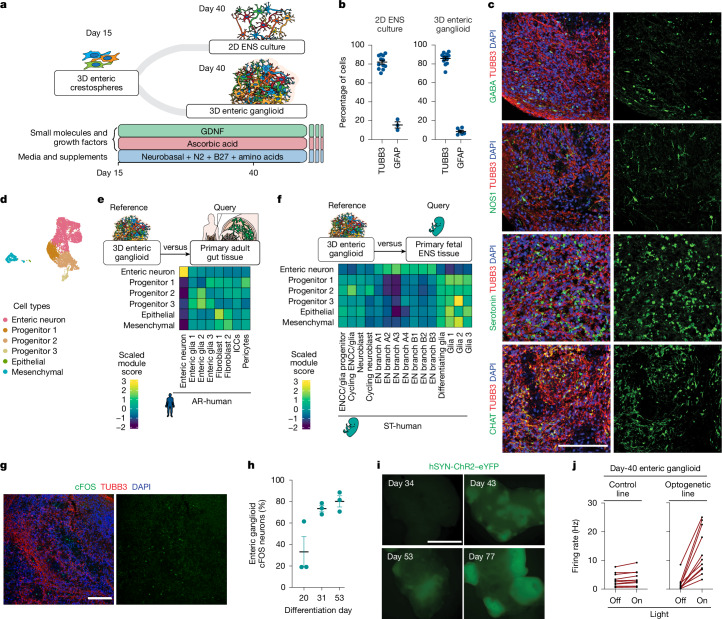
Development of hPSC-derived enteric ganglioids that model development, function and molecular diversity of human ENS. **a**, Protocol schematic for in vitro differentiation and maturation of hPSC-derived enteric crestospheres into 2D ENS cultures and 3D ganglioids. **b**, Flow cytometry quantification of neuronal (TUBB3) and glial (GFAP) markers in day-40 enteric ganglioids and 2D ENS cultures. Each dot represents a biologically independent differentiation experiment (data are the mean ± s.e.m.; left to right, *n* = 12, 3, 11 and 6). **c**, Immunofluorescence analysis for expression of ENS cell-type markers (serotonin, CHAT, GABA and NOS1) in day-40 enteric ganglioids. Imaging with neuronal subtype markers was performed for *n* = 3 biologically independent differentiation experiments. Scale bar, 100 μm. DAPI, 4′,6-diamidino-2-phenylindole. **d**, Single-nucleus RNA-sequencing uniform manifold approximation and projection of cell types present in day-40 enteric ganglioids; sample is a suspension pool of *n* = 5–10 ganglioids. **e**, Heat map of the average module scores of adult human colon cell-type transcriptional signatures in day-40 ganglioid cell type. ICCs, interstitial cells of Cajal. **f**, Heat map of the average module scores of ST-human ENS cell-type transcriptional signatures in hPSC-derived ENS progenitors, neurons and glia. **g**, Immunofluorescence analysis for expression of neuronal activity marker cFOS in day-40 and day-80 enteric ganglioids. Staining and imaging was performed for *n* = 3 biologically independent differentiation experiments. Scale bar, 100 μm. **h**, Flow cytometry quantification of neuronal activity marker cFOS in enteric ganglioids as they mature. Data are the mean ± s.e.m. of *n* = 3 biologically independent differentiation experiments. **i**, Live fluorescence images of human hSYN-ChR2–eYFP in enteric ganglioids as they mature. Imaging was performed for *n* = 3 biologically independent differentiation experiments. Scale bar, 1 mm. **j**, Quantification of MEA analysis of baseline and blue-light-stimulated neuronal activity in control (left) and day-40 hSYN-ChR2–eYFP (right) enteric ganglioids. Each dot represents an electrode recording; *n* = 11 electrodes, *n* = 3 ganglioids.

To determine the potential of enteric crestospheres to differentiate into ENS cell types, we established 2D and 3D culture conditions that facilitate the transition of ENCCs into ENS cell types (Fig. 1a). Both differentiation systems were highly efficient, yielding >80% TUBB3^+^ neurons (Fig. 1b). In addition, we identified a population of enteric glial precursors expressing GFAP and other glial lineage markers (Fig. 1b and Extended Data Fig. 1h). Although 2D cultures offer unique technical advantages, such as compatibility with quantitative imaging assays, we primarily focused on 3D cultures, referred to as enteric ganglioids. These models are more scalable and easier to maintain in long-term cultures. In addition, 3D culture systems offer practical advantages for applications such as cell therapy by eliminating the need for harsh cell dissociation steps before transplantation.

Enteric neurons are often classified on the basis of their neurochemical properties, including nitrergic, cholinergic, glutamatergic, catecholaminergic, GABAergic or serotonergic. We first verified the presence of neurons with various neurochemical features in our ganglioids and 2D ENS cultures by immunostaining (Fig. 1c and Extended Data Fig. 1i).

To define the cellular composition of enteric ganglioids more comprehensively, we performed single-nucleus RNA sequencing on day 40 of differentiation (Fig. 1a). Unbiased clustering revealed a large population of enteric neurons, three progenitor populations and small populations of contaminating epithelial cells and mesenchymal cells (Fig. 1d and Extended Data Fig. 1j). Lineage analysis using module scoring revealed that the progenitor 1 population shared high transcriptional similarity to ENCC 2′ and 4′. Furthermore, the mesenchymal population was highly similar to ENCC 3′ (Extended Data Fig. 1k). We confirmed the expression of all main neurotransmitter markers in our enteric neurons as shown in Extended Data Fig. 1l.

To assess whether hPSC-derived ENS cell populations recapitulate the molecular properties of the primary ENS, we utilized published primary human ENS (AR-human^18^ (Extended Data Table 1m) and ST-human^19^ (Extended Data Table 1n)). Module scoring of ganglioid cell-type signatures on primary cell types demonstrated that in vitro and in vivo enteric neurons were highly similar (Fig. 1e). Similarly, scoring the expression of hPSC-derived ENS cell population modules in primary human developing ENS cell types annotated in ref. ^19^ revealed similarity of day-40 progenitors to cycling ENCCs or glia (ENCC/glia), and hPSC-derived enteric neuron modules were expressed at high levels in the primary human enteric neuron subtypes (Fig. 1f). Non-neuron clusters were more similar to the primary glial clusters (Fig. 1f).

**Extended Data Fig. 2:**
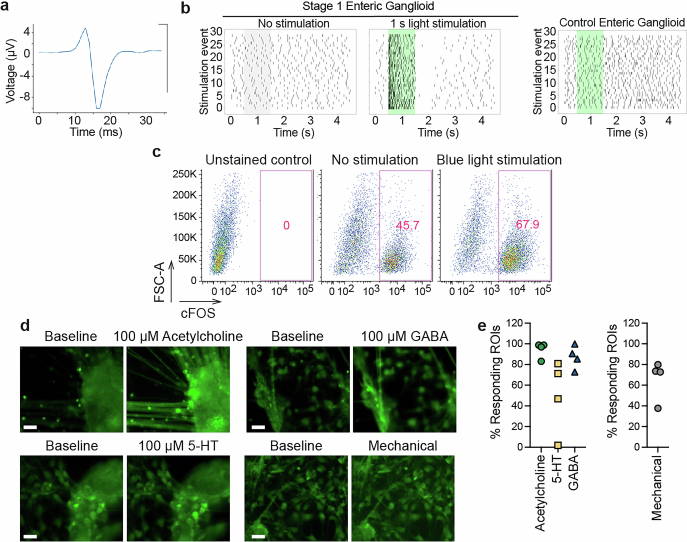
hPSC-derived enteric ganglioids comprise functionally active neurons. **a**) Representative diagram of a spontaneous neuronal firing recorded during MEA analysis of day 40 enteric ganglioids. **b**) MEA analysis of baseline and blue light-stimulated neuronal activities in day 40 hSYN-ChR2–EYFP (left) and control (right) enteric ganglioids. **c**) Flow cytometry analysis of cFOS expression in hSYN-ChR2–EYFP-derived day 80 enteric ganglioids in response to blue light stimulation. **d**,**e**) Representative images (**d**) and quantitative analysis (**e**) of real-time intracellular calcium dynamics in live day 50 enteric neuron cultures following neuromodulator stimulation. Scale bar = 50 uM.

To assess functional maturation, we measured cFOS, a well-established marker of neuronal activity induced by depolarization^20–22^. cFOS expression increased as the enteric ganglioids progressed during differentiation (Fig. 1g,h). To demonstrate synaptic maturation and electrical excitability of enteric neurons within ganglioids, we used optogenetics by differentiating a reporter human embryonic stem cell (hESC) line that expresses enhanced yellow fluorescent protein (eYFP)-tagged channelrhodopsin-2 (ChR2) under control of the human synapsin (hSYN) promoter^23^. eYFP was readily detectable as early as day 43 (Fig. 1i). Light stimulation of ganglioids increased electrical firing rates, as detected by microelectrode array (MEA; Fig. 1j and Extended Data Fig. 2a,b), leading to increased cFOS expression as compared to unstimulated enteric ganglioids (Extended Data Fig. 2c). Thus, hPSC-derived enteric neurons are functional and continue to gain maturity over time. To evaluate the responsiveness of hPSC-derived enteric neurons to external stimuli, we performed live-cell calcium imaging. Stimulation with the neuromodulators acetylcholine, GABA and serotonin elicited a rise in cytosolic calcium, visualized using the fluorescent indicator Fluo-4 AM (Extended Data Fig. 2d,e). Notably, the neurons exhibited strong responses to neurotransmitters, particularly acetylcholine, as well as a distinct calcium influx in response to mechanical stimulation induced by the rapid injection of a small volume of water into the culture medium (Extended Data Fig. 2d,e).

These findings provide a framework for accessing scalable hPSC-derived enteric neuron cultures that recapitulate the key molecular and functional characteristics of primary enteric neurons.

## Ganglioids contain enteric NO neurons

GI motility is regulated by enteric excitatory and inhibitory motor neurons, with a major subset of inhibitory neurons using NOS1 to produce NO, which relaxes smooth muscle and maintains mucosal integrity^24–27^. NO neurons are central to motility control, and their selective loss or dysfunction is linked to disorders such as achalasia, gastroparesis, intestinal pseudo-obstruction and colonic inertia^1,2^.

**Fig. 2:**
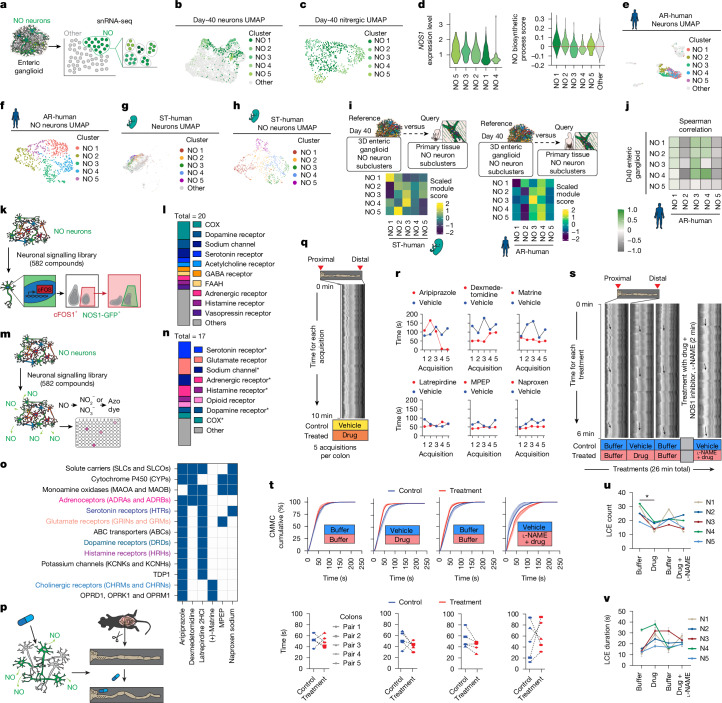
NO neuron functional screening identifies colonic motility modulators. **a**–**c**, Single-nucleus RNA-sequencing (snRNA-seq) workflow (**a**), uniform manifold approximation and projection (UMAP) of NO neurons (**b**) and subtypes (**c**) in enteric ganglioids. **d**, Violin plot of *NOS1* expression and module scoring for NO biosynthesis genes by enteric ganglioid NO neuron subtypes. **e**–**h**, UMAP of NO neurons (**e**,**g**) and subtypes (**f**,**h**) in AR-human (**e**,**f**) and ST-human (**g**,**h**). **i**, Heat map of average module scores of ganglioid NO neuron subtype signatures in ST-human and AR-human. **j**, Spearman correlation of AR-human versus ganglioid NO subtypes. **k**,**l**, Schematic of high-throughput flow cytometry screen to identify compounds inducing cFOS in enteric ganglioid NO neurons (**k**), and target classes of resulting hits (**l**; Extended Data Fig. 4g). Cells from *n* = 5 differentiations were pooled for *n* = 1 screen. **m**,**n**, Schematic of high-throughput colorimetric screen to identify compounds inducing NO release in 2D ENS cultures (**m**) and target classes of the hits (**n**; Extended Data Fig. 4h). Asterisks mark protein classes also in **j**. Cells from *n* = 5 differentiations for *n* = 1 screen. **o**, Combined protein target analysis for selected hits. Target colour codes match **l** and **n**. **p**, Schematic of testing candidate compounds (from **o**) on mouse colonic motility ex vivo. **q**, Representative spatiotemporal map of mouse colonic contractions along the proximal–distal axis over 10 min. **r**, Quantification of CMMC intervals at the 75th percentile of cumulative CMMC distribution (Extended Data Fig. 5a) for each compound (*n* = 1 mouse pair per compound, 5 recordings per pair). **s**, Schematic and representative 26-min spatiotemporal maps; arrows indicate three LCEs per condition. **t**–**v**, Quantification of CMMC intervals at the 75th percentile (**t**), total LCEs per 6-min segment (**u**) and mean LCE duration (**v**, based on early, mid and late events); *n* = 5 dexmedetomidine-treated versus untreated colon pairs, mean ± s.e.m. One-way analysis of variance (ANOVA) with Dunnett’s test in **u**, *P = 0.0208 < 0.05. Source data

**Extended Data Fig. 3:**
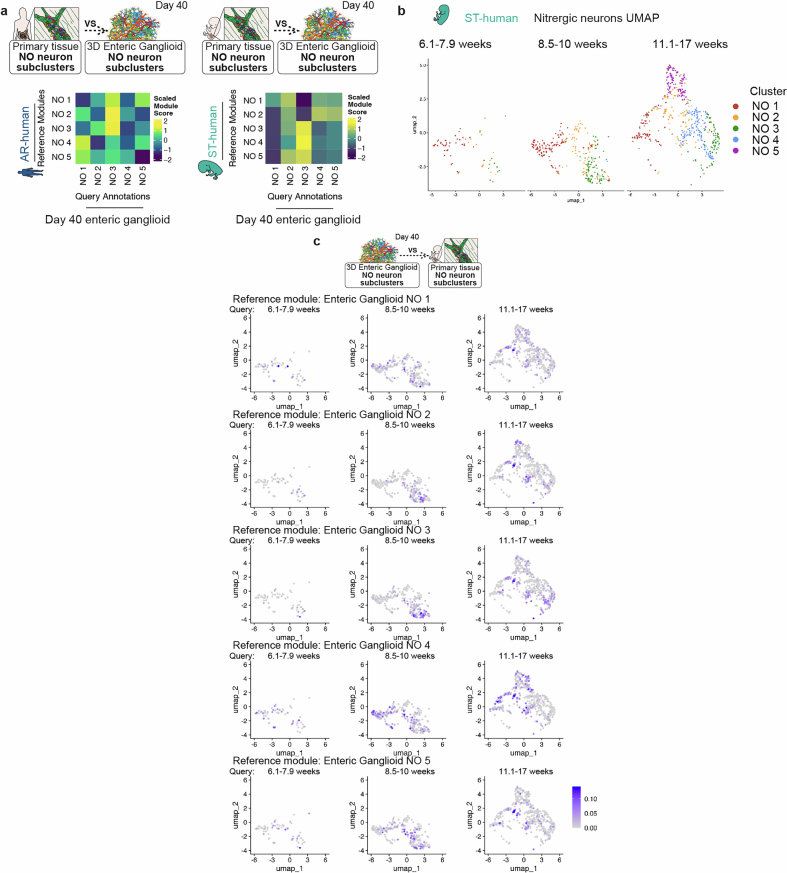
Enteric ganglioids NO neuron transcriptional signatures are present throughout human fetal development. **a**) Heatmap of the average module scores of AR-human (left) and ST-human (right) NO neuron subtype transcriptional signatures in day 40 enteric ganglioid NO neuron subtypes. **b**) Split UMAP split plots of ST-human NO neuron subtypes from different embryonic developmental timepoints. **c**) Feature plots showing the expression of enteric ganglioid NO neuron subtype transcription signature modules by ST-human NO neurons in human developmental timepoints.

Our hPSC-derived cultures comprise a diverse neuronal population including NO neurons. Having access to this subtype of neurons prompted us to perform deeper characterization of their molecular and functional identities and develop assays to understand and modulate their activity. In single-nucleus RNA-sequencing datasets of our day-40 enteric ganglioids, the NO neurons were identified by their expression of the key marker gene, *NOS1*, and selected metabolic and NO transport genes (Fig. 2a,b). To determine the molecular diversity within the NO neurons, we performed further subclustering and identified five subtypes (NO 1–5; Fig. 2b,c). In addition to *NOS1* expression, these clusters showed enrichment for other NO biosynthesis pathway genes, confirming their shared NO identity (Fig. 2d). Similar to that for enteric ganglioids, subclustering NO neurons in the adult and embryonic human primary ENS dataset generated in refs. ^18,19^ identified five primary NO neuron subtypes (AR-human NO 1–5; ST-human NO 1–5; Fig. 2e–h). Module scoring revealed similarities between hPSC-derived and primary human NO neuron subtypes (Fig. 2i and Extended Data Fig. 3a). Likewise, Spearman correlation analysis based on the expression of 3,000 anchor features showed correlations for NO 2–5 with AR-human NO 3, as well as NO 2, 4 and 5 with AR-human NO 4 (Fig. 2j). We observed that ST-human NO neurons from clusters 1, 2 and 3 were present across all human developmental time points (Extended Data Fig. 3b). By contrast, NO neurons from clusters 4 and 5 were predominantly detected in the 11.1–17-week time point (Extended Data Fig. 3b). Module scoring of ganglioid NO neuron signatures in human fetal NO neurons across developmental time points revealed the presence of all subtype signatures at every stage analysed (Extended Data Fig. 3c). This finding suggests that NO neuron clusters in ganglioids do not correspond to distinct maturation states but may instead reflect functional diversity.

## ENS models identify motility enhancers

Given the important role of enteric NO neurons in GI physiology and disease^1,2^, there has been a great deal of interest in establishing strategies to regulate their function. Factors that modulate NO neuron activity and increase NO release could serve as potential therapeutic targets. Hence, we leveraged our scalable ENS culture platforms to screen for drug candidates that induce NO neuron activity.

**Extended Data Fig. 4:**
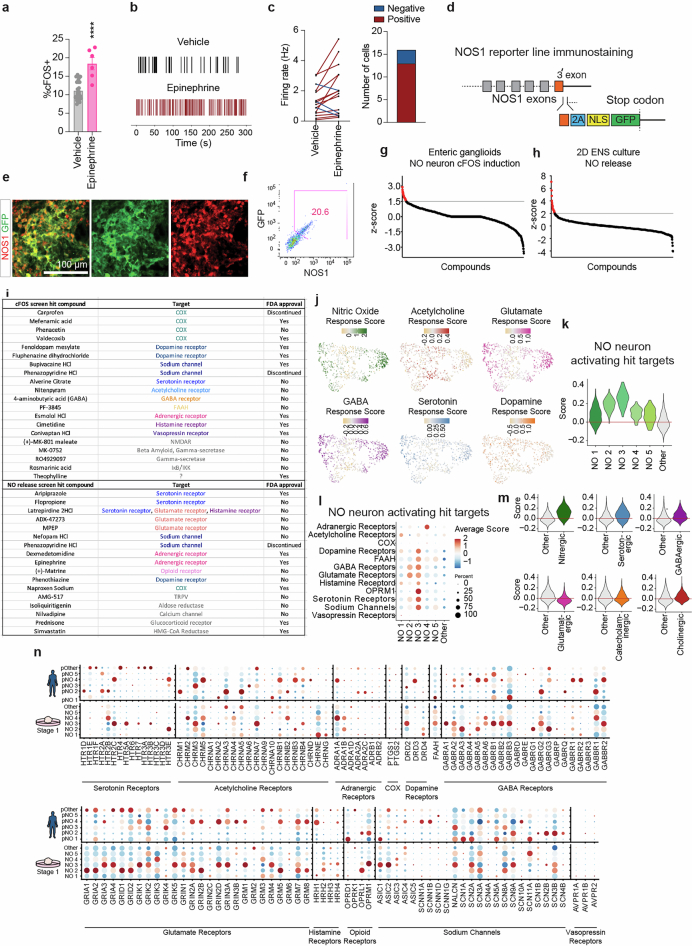
Identifying enteric NO neuron modulators by functional high-throughput screenings. **a**) Flow cytometry quantification of enteric ganglioid cFOS expression in response to epinephrine. Mean and SEM error bars are shown, ****: p-value < 0.0001. **b**,**c**) MEA analysis (**b**) and quantification of changes in neuronal firing (**c**) in response to epinephrine in day 40 enteric ganglioids. **d**) Schematic representation of the NOS1-GFP reporter construct. **e**) Representative immunofluorescence images of a enteric ganglioid stained for GFP and NOS1. **f**) Representative flow cytometry analysis for the expression of GFP and NOS1 in a NOS1-GFP-derived enteric ganglioid. **g**) Identifying candidate neuromodulators that induce cFOS expression in enteric ganglioid NO neurons by HTS. **h**) Identifying candidate neuromodulators that induce NO release in day 40 2D ENS culture NO neurons by HTS. **i**) Predicted targets and FDA approval status of hits identified in cFOS induction and NO release screens. **j)** Feature plots showing the predicted responsiveness of subclustered stage 1 ganglioid enteric NO neurons to neurotransmitters by module scoring of neurotransmitter receptor gene families. **k**) snRNA-seq analysis violin plot of module scoring for all predicted NO neuron activity promoting receptor genes in day 40 enteric ganglioid NO subtypes versus other neurons. **l**) snRNA-seq analysis dot plot of the average module scores for individual categories of NO neuron activity promoting receptor gene categories. **m)** snRNA-seq analysis violin plot of module scoring for all predicted NO neuron activity promoting receptor genes in stage 1 enteric ganglioid neurotransmitter subtypes versus other neurons. **n)** Dot plot of the expression of genes belonging to the target classes shown in **2l** and **2n** in hESC-derived stage 1 and primary human enteric nitrergic neuron subtypes versus all other neurons.

We screened for NO neuron activity using cFOS expression as a read-out. To evaluate cFOS as an accurate read-out of neurochemical-induced activity, we performed side-by-side cFOS flow cytometry analysis and MEA neuronal firing measurements in cultures treated with adrenaline, which is known to stimulate enteric neurons. Adrenaline induced neuronal cFOS expression and resulted in increased electrical firing of ganglioid neurons. This provides a scalable read-out of activity that is suitable for high-throughput screens (Extended Data Fig. 4a–c).

To facilitate the labelling and detection of NO neurons in the screen, we generated a hESC NOS1-GFP line by inserting a *GFP* cassette under control of the endogenous *NOS1* promoter using a CRISPR–Cas9 knock-in technique (Extended Data Fig. 4d). Following our ENS induction protocol, NOS1-GFP hESCs gave rise to mature cultures with NO neurons co-expressing GFP and NOS1 (Extended Data Fig. 4e,f). We performed a cFOS induction screen, in which NOS1-GFP-containing enteric ganglioids were acutely exposed (1 h) to a library of 582 neuromodulators and co-expression of cFOS and GFP was measured to quantify NO neuron activity (Fig. 2k and Extended Data Fig. 4g). We identified 20 compounds that increased the proportion of NO neurons in cFOS^+^ cells with *z*-score > 1.5. To identify the mechanisms involved in cFOS expression in NO neurons, we compiled and classified the list of target proteins and discovered multiple shared protein classes (Fig. 2l).

In a second complementary functional screen, we established a high-throughput read-out for assessing NO neuron activity. We utilized a commercially available assay system that enables NO detection in the medium. We incubated ENS cultures with the neuromodulator library and measured NO release using colorimetry (Fig. 2m). For this assay, we used 2D ENS cultures, which are technically compatible for plate-based colorimetric assays. We identified 17 compounds that remarkably enhanced NO concentration in the supernatant with *z*-score > 2.0 (Extended Data Fig. 4h). Neuromodulators that induced NO release in our ENS cultures were diverse but were predicted to target common target classes (Fig. 2n).

We found substantial overlap between predicted targets from the cFOS induction and NO release screens (Fig. 2l,n and Extended Data Fig. 4i). These targets included receptors for neurotransmitters such as serotonin and dopamine. Module scoring the neurotransmitter receptor gene families in our hPSC-derived enteric NO neurons single-nucleus RNA-sequencing data confirmed that NO neurons broadly express receptors for NO, serotonin, GABA, glutamate, acetylcholine or dopamine (Extended Data Fig. 4j). Module scoring of the predicted hit targets showed the highest enrichment score in NO neuron clusters compared to other neuronal subtypes (Extended Data Fig. 4k,l). We also observed enriched expression of this module in serotonergic and GABAergic neurons (Extended Data Fig. 4m). In particular, the NO 3 cluster scored high for the expression of most NO neuron modulator target classes (Extended Data Fig. 4k,l). Profiling the expression of individual genes in each target protein category in ganglioids and primary human ENS revealed notable subtype-specific expression patterns between NO neurons. For example, GABA receptor genes were predominantly expressed by NO 2, NO 3 and AR-human NO 4 subtypes, whereas the expression of acetylcholine receptor genes was less specific to a particular subtype (Extended Data Fig. 4n).

**Extended Data Fig. 5:**
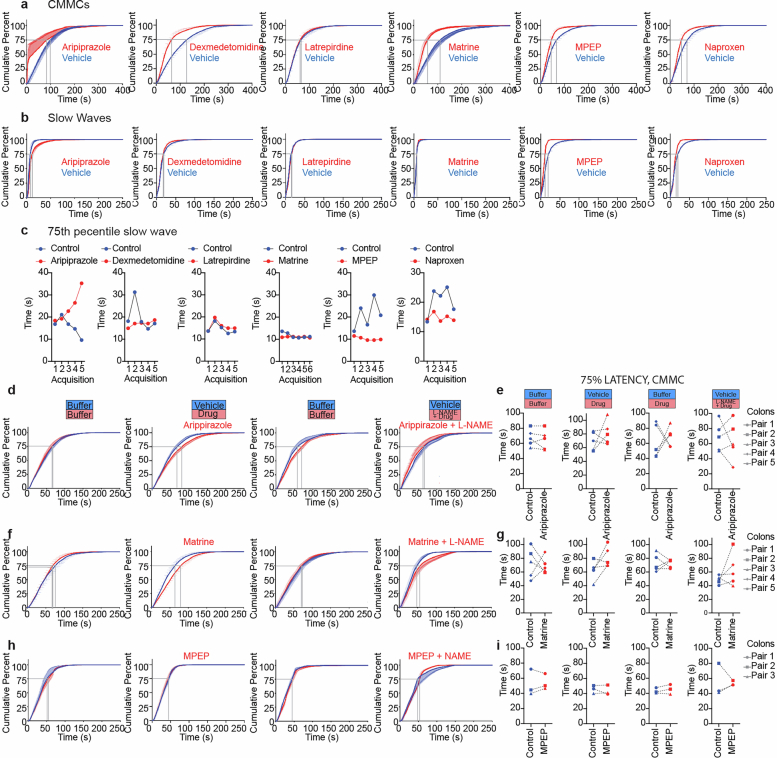
Effect of candidate drugs in ex vivo mouse colonic motility assays. **a**,**b**) Diagrams of CMMC (**a**) and slow wave (**b**) cumulative percentiles. **c**) Quantification of latency between slow waves at 75^th^ percentile for selected HTS hits; from left to right: aripiprazole, dexmedetomidine, latrepirdine, matrine, MPEP and naproxen. One-directional SEM error bars are shown in panels **a** and **b**. **d**,**i**) Diagrams of CMMC cumulative percentile and quantifications of CMMC intervals at 75^th^ percentile for aripiprazole (**d**,**e**), matrine (**f**,**g**), and MPEP (**h**,**i**). Compounds were used at 1 μM and mean and one-directional SEM error bars are shown in panels **d**,**f**,**h**. Source data

**Extended Data Fig. 6:**
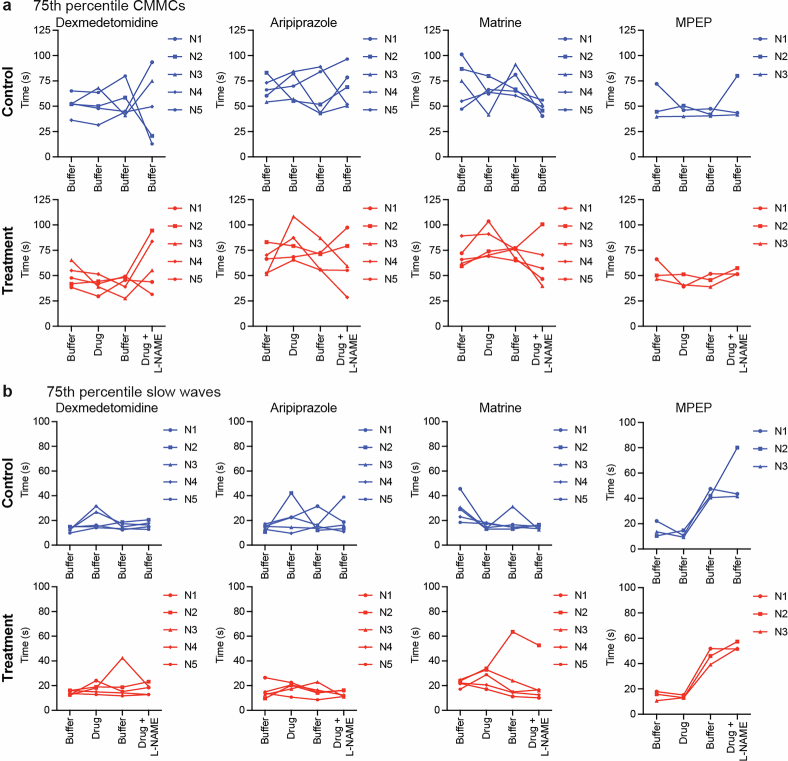
Effect of candidate drugs on ex vivo mouse colonic CMMC and slow wave intervals at 75^th^ percentile. **a**,**b**) Quantifications of CMMC (**a**) and slow wave (**b**) intervals (time difference between two consecutive contractions) at 75^th^ percentile for dexmedetomidine, aripiprazole, matrine, and MPEP. In each panel, top rows (blue) show control colons and bottom rows (red) show treatment colons within each pair. Compounds were used at 1 μM. Source data

**Extended Data Fig. 7:**
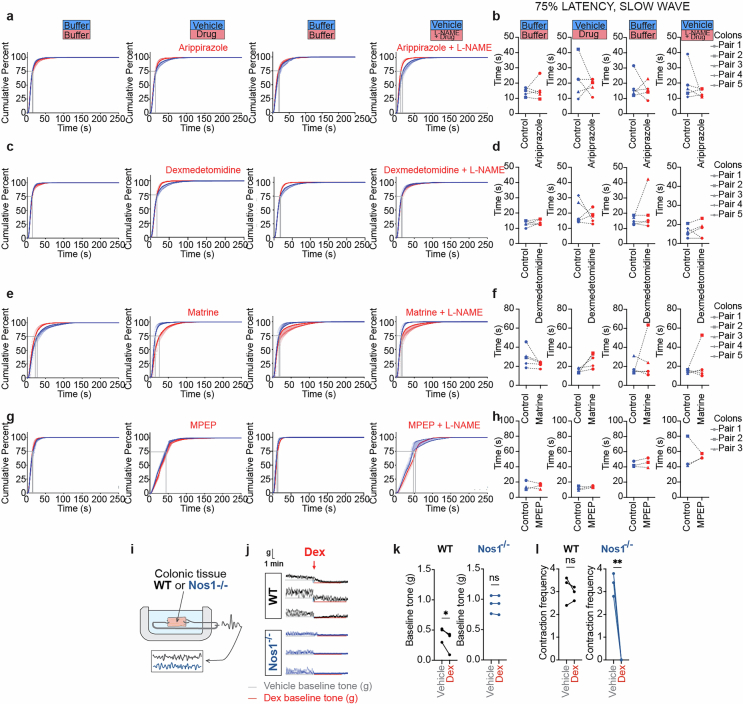
Effect of candidate drugs on ex vivo mouse colonic slow waves. **a-h**) Diagrams of slow wave cumulative percentile and quantifications of latency between slow waves at 75^th^ percentile for aripiprazole (**a**,**b**), dexmedetomidine (**c**,**d**), matrine (**e**,**f**), and MPEP (**g**,**h**). Compounds were used at 1 μM and mean and one-directional SEM error bars are shown in panels **a**, **c**, **e** and **g**. **i**) Schematic representation of contractility monitoring organ bath system used for analyzing the impact of dexmedetomidine on pieces of colonic tissue from WT and Nos1-/- mice. Schematic adapted from reprocell.com. **j**) Contraction across time traces recorded from tissue samples derived from 3 WT and 3 Nos1-/- mouse colons. Horizontal lines indicate basal tone/minimal point in vehicle (gray) and dexmedetomidine (red) conditions. **k**) Quantification of basal tone, one-tailed paired t-test was used for analysis. *: p-value < 0.05. **l**) Quantification of average contraction frequency, two-tailed paired t-test was used for analysis. **: p-value < 0.01. Source data

We then selected a subset of hits representing different target classes, prioritizing US Food and Drug Administration (FDA)-approved compounds for follow-up analyses (Fig. 2o and Extended Data Fig. 4i). For selected compounds, we performed a more comprehensive and integrated target analysis by combining reported experimental binding assay data (BindingDB) and computational methods (SEA, Carlsbad, Dinies, Swisstarget, Superdrug and Pubchem Bioassays; Fig. 2o). We next tested the effects of these compounds on colonic motility in ex vivo organ bath assays (Fig. 2p). In the first experiment, we tested the effect of all selected drug candidates on mouse colonic motility, ex vivo (Fig. 2q). In each experiment, we tested an untreated control and a drug-treated colon sample side by side during five consecutive 10-min acquisitions. For each acquisition, we generated spatiotemporal maps from video data based on the changes in the colonic diameter over time and used them to calculate the rate of colonic migrating motor complexes (CMMCs) and slow waves (SWs). CMMCs are rhythmic propulsive contractions initiated by the ENS, whereas SWs are mediated through the pacemaking activity of interstitial cells of Cajal^28–32^. To probe the dynamics of CMMC and SW events during each acquisition, we generated cumulative percentage graphs (Extended Data Fig. 5a,b) and calculated the intervals at the 75th percentile (Fig. 2r and Extended Data Fig. 5c). Compounds that showed promising effects on shortening CMMC intervals relative to the untreated condition were chosen for follow-up assessment (that is, aripiprazole, dexmedetomidine, matrine and MPEP; Fig. 2r and Extended Data Fig. 5a–c). To assess whether the compounds mediated their effect on CMMCs through modulating NO release, we performed sequential drug treatments in the presence and absence of the NOS1 inhibitor *N*ω-nitro-l-arginine methyl ester (l-NAME). Each experiment consisted of four 6-min acquisitions on five independent sample pairs in the control and drug-treated groups (Fig. 2s). Of the tested drugs, the adrenergic receptor agonist dexmedetomidine decreased CMMC intervals in four out of five colon samples, an effect that was absent when colons were treated with dexmedetomidine and l-NAME simultaneously (Fig. 2t, Extended Data Figs. 5d–i and 6a,b and Supplementary Video 1). SWs were not affected by the drug treatment (Extended Data Fig. 7a–h).

In addition to CMMCs, we quantified the effects of compounds on anterograde contractile events detected in the spatiotemporal map. These events were termed longitudinal contractile events (LCEs) and are highlighted by representative arrows in Fig. 2s. In dexmedetomidine-treated colons, we observed a decrease in the total number of LCEs (Fig. 2u) and an increasing trend in their average duration (Fig. 2v) in all five replicates. These effects were reversed after the drug removal and blocked by l-NAME co-treatment (Fig. 2u,v).

To further investigate dexmedetomidine’s response on NO neurons, we used an alternative, previously described^33^, organ myobath assay that enabled measuring the mechanical properties of isolated colonic tissues from wild-type and *Nos1*^*−/−*^ mice (Extended Data Fig. 7i). Dexmedetomidine application to wild-type tissues reduced baseline tone, suggesting relaxation (Extended Data Fig. 7j,k). However, dexmedetomidine was unable to reduce baseline tone in *Nos1*^*−/−*^ tissues (Extended Data Fig. 7j,k). The reduced tensile activity in the wild-type tissues was not linked to changes in contraction frequency, which remained unaltered in wild-type tissues, whereas contraction was abolished in *Nos1*^*−/−*^ tissues (Extended Data Fig. 7l). Although dexmedetomidine’s impact on the enteric neural circuit probably involves additional mechanisms, these experiments strongly indicate a notable effect of the drug candidate on colonic muscle tone through nitrergic activity.

These results provide a blueprint for leveraging in vitro human ENS models to uncover mechanisms that regulate GI motility that are capable of identifying therapies that target specific ENS populations.

## PDGFR inhibitors drive NO neuron fate

**Fig. 3:**
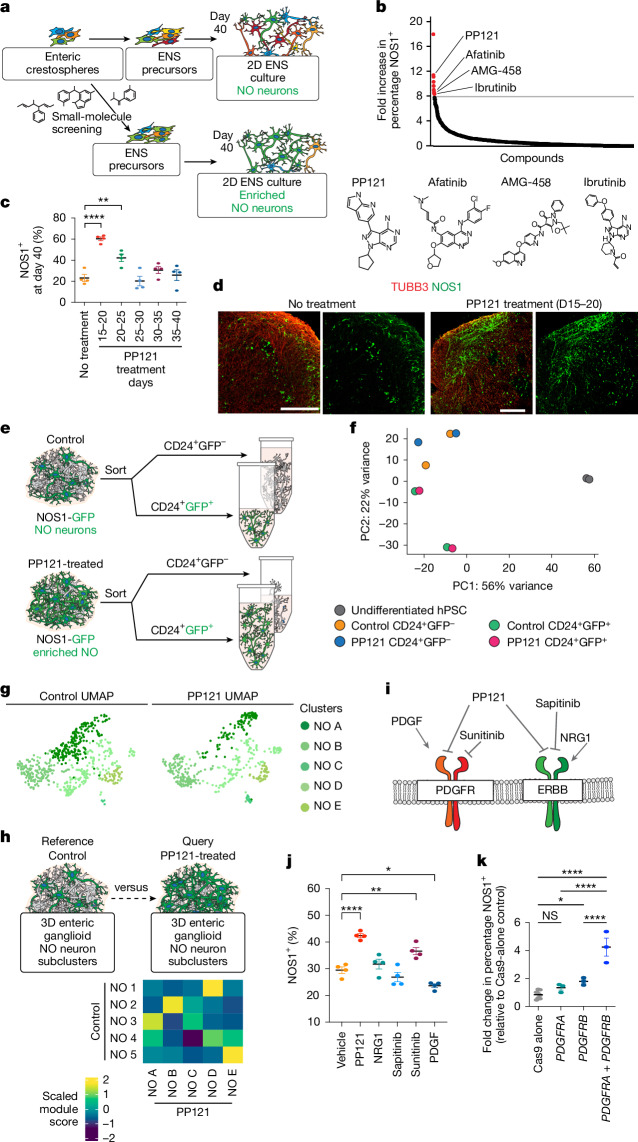
PDGFR inhibition promotes enteric NO neuron induction. **a**, Schematic representation of a high-throughput pharmacological screening procedure to identify compounds that enrich NO neurons in 2D ENS cultures derived from hESCs. **b**, Identifying candidate compounds that enrich NO neurons in day-40 2D ENS cultures. *n* = 1 high-throughput library screening procedure. **c**, Effect of PP121 treatment window on NOS1-GFP induction efficiency. *n* = 4 biologically independent ganglioid differentiations, mean ± s.e.m, one-way ANOVA with Dunnett’s multiple comparisons, **P < 0.01 and ****P < 0.0001. **d**, Immunofluorescence staining of NOS1 and neuronal TUBB3 in day-40 enteric ganglioids treated with or without PP121 between days 15 and 20. Scale bars, 100 μm. **e**, Schematic of the fluorescence-activated cell sorting experiment using NOS1-GFP reporter line day-40 enteric ganglioids, sorting CD24^+^GFP^+^ (NOS1^+^ neurons) and CD24^+^GFP^−^ (NOS1^−^ neurons). **f**, Principal component analysis plot showing the transcriptional profiles of the sorted samples in **e**. Two biological replicates per sample were analysed. **g**, Split UMAP of subclustered NO subtypes present in day-40 control (left) and PP121-treated (right) enteric ganglioid cultures. **h**, Heat map of the average module scores of control-alone NO neuron subtype transcriptional signatures in PP121-treated ganglioid NO neuron subtypes. **i**, Schematic of RTK natural agonists and selected pharmacological antagonists including NO neuron enriching top hit PP121. **j**, Effect of RTK ligand treatment on day-40 enteric ganglioid NO neuron induction. *n* = 4 biologically independent ganglioid differentiations, mean ± s.e.m, one-way ANOVA with Dunnett’s multiple comparisons, *P < 0.05, **P < 0.01, ****P < 0.0001. **k**, Effect of knocking out *PDGFRA*, *PDGFRB* or both in day 15 enteric crestospheres on day-40 enteric ganglioid NO neuron enrichment as measured by flow cytometry. Dots show biologically independent knockout experiments (*n* = 6 control, *n* = 3 others), mean ± s.e.m, one-way ANOVA with Dunnett’s multiple comparisons, *P < 0.05, ****P < 0.0001; NS, not significant.

**Extended Data Fig. 8:**
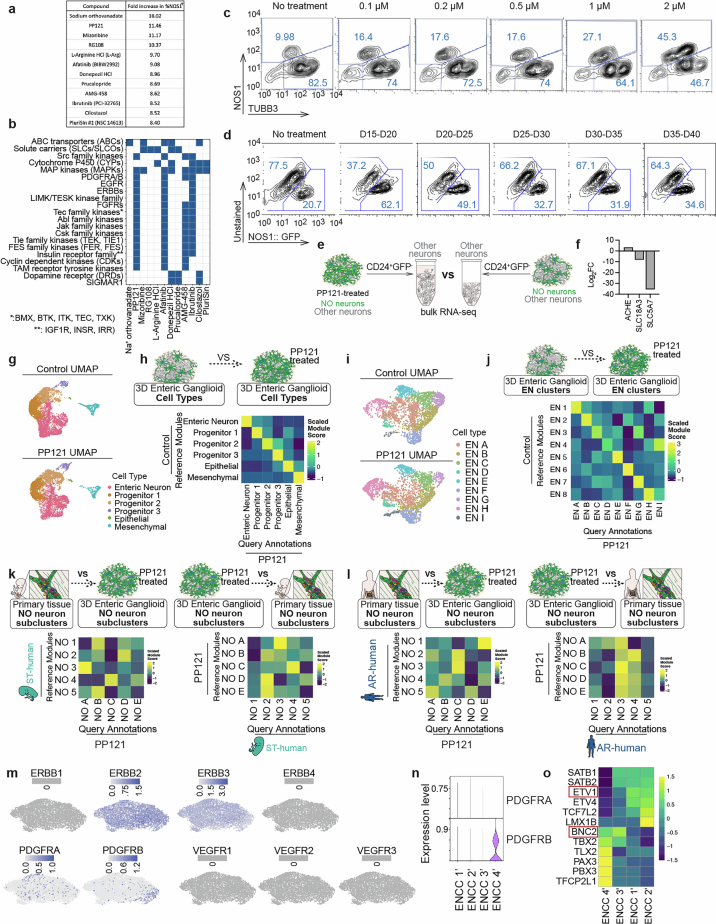
High-throughput screening identifies compounds that enrich NO neurons in hESC-derived ENS cultures. **a**) HTS hits with a > 8.0 fold increase in %NOS1^**+**^ cells. **b**) Combined protein target analysis revelas shared protein classes among top 12 structurally similar hits. **c**,**d**) PP121-induced NOS1 expression is dose- and time-dependent. Representative flow diagrams showing NOS1 expression with varying PP121 concentrations (**c**, Day 15–Day 20) and treatment windows (**d**, 2 μM). **e**,**f**) Schematic of the sorting strategy used for the bulk RNA-sequencing comparing non-NO neurons of enriched NO and control enteric ganglioids (**e**). Bulk RNA-sequencing (**f**) showing differentially expressed acetylcholine synthesis and release markers in non-NO neurons (CD24-GFP-) isolated from PP121 treated and control ganglioids. **g**) Split UMAP of cell types in day 40 control (top) and PP121-treated (bottom) ganglioids. **h**) Heatmap of the average module scores of control only enteric ganglioid subtype transcriptional signatures in PP121 treated ganglioidl subtypes. **i**) Split UMAP of neuronal subtypes in control (top) and PP121 treated (bottom) day 40 enteric ganglioids. **j**) Heatmap of the average module scores of control only neuronal subtype transcriptional signatures in PP121 treated ganglioid neuronal subtypes. **k**,**l**) Module score heatmaps of ST-human (**k**) and AR-human (**l**) NO subtype signatures in PP121-treated hPSC-derived NO neurons (left) and merged control + PP121 NO signatures in ST- or AR-human subtypes (right). **m**) Feature plot of ERBBs, PDGFRs and VEGFRs expression in D15 subclustered enteric crestospheres. **n**) Violin plots of PDGFRA and PDGFRB in crestosphere ENCC clusters. **o**) Heatmap of scaled expression of enteric neuron lineage transcription factor markers in crestosphere ENCC’ clusters.

To develop strategies to generate cultures enriched for NO neurons, we used a high-throughput small-molecule screen to identify the mechanisms of NO neuron differentiation. We treated enteric crestospheres with 1,694 small-molecule inhibitors and identified 12 hit compounds that increased the proportion of NOS1^+^ neurons by at least eightfold (Fig. 3a,b and Extended Data Fig. 8a). To reveal the mechanisms by which these hit compounds enhanced NO neuron induction, we performed target prediction analysis by combining reported experimental binding assay data (BindingDB) and computational methods (SEA, Carlsbad, Dinies, Swisstarget, Superdrug and Pubchem Bioassays; Extended Data Fig. 8b). After clustering the predicted protein targets, common patterns emerged for a subset of compounds. For example, PP121, ibrutinib, afatinib and AMG-458 were all predicted to interact with epidermal growth factor receptor (EGFR), ERBBs, and MAP and TEC family kinases among others (Extended Data Fig. 8b). Our high-throughput screening assay identified orthovanadate as the compound with the highest efficiency in inducing NOS1^+^ neurons. However, as orthovanadate is a phosphate analogue and a highly nonspecific inhibitor, we chose to follow-up on PP121, which had the second-highest overall fold increase in percentage of NOS1^+^ neurons. PP121 showed a dose-dependent effect on NO neuron induction efficiency as measured by flow cytometry (Extended Data Fig. 8c). To find the most effective treatment window for PP121-induced NO neuron induction, we treated the differentiating cultures for 5 days starting at various time points. Measuring GFP signal in NOS1-GFP enteric ganglioids at day 40 showed the highest induction efficiency for cells treated during day 15–20 of differentiation (Fig. 3c,d and Extended Data Fig. 8d).

For the enrichment protocol to be reliable, it was important to confirm that PP121 treatment did not change the identity of our cell types. Using the NOS1-GFP reporter line, we performed fluorescence-activated cell sorting using the neuronal surface marker CD24^+^ to isolate NO neurons (CD24^+^GFP^+^) and non-NO neurons (CD24^+^GFP^−^) for unbiased transcriptional comparisons using bulk RNA sequencing. Principal component analysis confirmed that PP121 treatment does not affect the transcriptional identity of NO neurons, even though it increases their proportion (Fig. 3e,f). Notably, PP121-treated cultures exhibited a reduction in cholinergic markers compared to control ganglioids, suggesting that the increased proportion of nitrergic neurons potentially occurs at the expense of cholinergic neurons (Extended Data Fig. 8e,f). To compare PP121-treated and untreated day-40 enteric ganglioids at single-cell resolution, we performed single-nucleus RNA sequencing and combined both datasets. This analysis revealed that all cell types were represented in both conditions (Extended Data Fig. 8g,h). Subclustering of the merged control and PP121-treated neurons revealed nine neuronal subtypes, enteric neuron (EN) A–I (Extended Data Fig. 8i). The subtypes in the dataset of PP121-treated neurons showed high transcriptional similarity to EN 1–8 of the control-alone dataset (Extended Data Fig. 8j). EN cluster I consisted of mostly PP121-treated cells and few control cells and showed moderate transcriptional similarity to the control-alone EN cluster 4, suggesting that this neuronal subtype is present but rare in control cultures, causing those neurons to cluster with the most similar subtype, EN 4 (Extended Data Fig. 8h). Further subclustering of merged control and PP121-treated nitrergic neurons revealed an enrichment for nitrergic B (most similar to control-alone nitrergic 2) and a rare population, nitrergic C (most similar to control-alone nitrergic 3; Fig. 3g,h). We next compared the gene expression profile of nitrergic neurons in PP121-treated ganglioids to that of primary human nitrergic neurons. Module scoring of NO neuron subtype signatures in PP121-treated ganglioids and primary human fetal and adult NO neurons revealed that PP121-treated ganglioids contained a diverse population of NO neurons with transcriptional similarities to their primary counterparts (Extended Data Fig. 8k,l). Altogether, these data suggest that early treatment of ganglioids with PP121 causes changes in the abundance of neuronal subtypes normally found in untreated cultures without affecting the gene expression patterns of the subtypes that arise.

To determine the mechanism by which PP121 induced NO neuron enrichment, we used a combination of pharmacological and genetic approaches. PP121 is a multi-targeted receptor tyrosine kinase (RTK) inhibitor with known inhibitory activity on PDGFRs, vascular endothelial growth factor receptors (VEGFRs) and EGFRs^34^. Our single-nucleus RNA-sequencing analysis confirmed the expression of PDGFRA, PDGFRB, ERBB2 and ERBB3 in our progenitor cultures (crestospheres), whereas the mRNAs for VEGFRs were not detectable (Extended Data Fig. 8m). We evaluated the induction efficiency of NO neurons in response to PDGF (PDGFR agonist), sunitinib (PDGFR and VEGFR antagonist), NRG1 (agonist of ERBBs) and sapitinib (antagonist of ERBBs; Fig. 3i). Whereas NRG1 and sapitinib showed no significant effect on NO neuron induction, treatment with PDGF and sunitinib led to lower and higher NO neuron proportions, respectively (Fig. 3j). Single-nucleus RNA-sequencing analysis showed a high level of expression of PDGFRA and PDGFRB transcripts in crestosphere’s ENCC 4′ population (Extended Data Fig. 8n). We next assessed the expression of transcription factors known to be involved in enteric neuron diversification and identified distinct expression patterns, including a clear separation between ETV1 and BNC2 expression in ENCC clusters, consistent with previous observations^35–37^. Notably, BNC2 expression, previously associated with the cholinergic lineage^35^, was higher in ENCC 4′, whereas ETV1 expression, linked to the nitrergic neuron lineage^35^, was elevated in other ENCCs, which showed a low level of PDGFR expression (Extended Data Fig. 8n,o).

To further confirm the role of PDGFR signalling in NO neuron induction, we used CRISPR–Cas9 to knockout *PDGFRA*, *PDGFRB* or both in our enteric crestospheres and analysed the percentage of NO neurons in ganglioids. NO neurons were enriched in both *PDGFRA*- and *PDGFRB-*knockout cultures and to a higher extent in the double-knockout cells, confirming the role of these receptors in PP121-mediated increase in NO neuron induction (Fig. 3k).

## Transplanted ganglioids improve motility

**Fig. 4:**
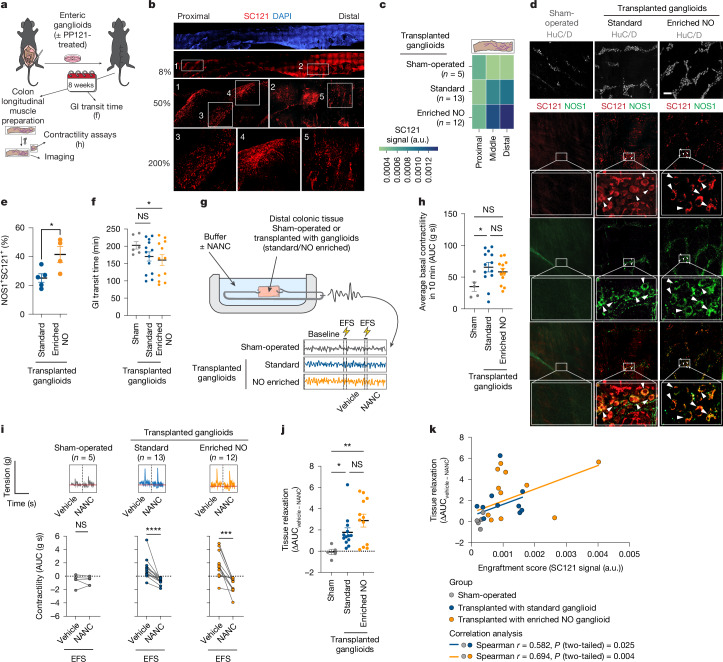
Extensive engraftment of hESC-derived enteric ganglioids in adult mouse colon. **a**, Schematic of transplantation of enteric ganglioids derived from hESCs into mouse distal colon. **b**, Engraftment of enteric ganglioid cells across the mouse colon (human cytoplasmic marker SC121 (red)). **c**, Schematic and quantification of SC121 signal in distal colon from sham and transplanted *Nos1*^*−/−*^ mice; data reflect average signal from *n* = 5 regions of interest from each colon. a.u., arbitrary units. **d**, Immunofluorescence images showing neuronal marker (HuC/D), SC121 and NOS1 in distal colon tissues from sham and transplanted animals. Arrow heads point at transplanted NO neurons (SC121^+^NOS1^+^). Scale bar, 100 μm. **e**, NOS1^+^ neuron frequency in *Nos1*^*−/−*^ colons transplanted with standard versus NO-enriched ganglioids. *n* = 4–5 mice, mean ± s.e.m. Non-parametric one-tailed unpaired *t*-test (Mann–Whitney test) was used for analysis, *P = 0.0159 < 0.05. **f**, GI transit time in sham-operated and transplanted *Nos1*^*−/−*^ mice. Each dot shows a biologically independent animal (*n* = 6 sham, *n* = 13 standard, *n* = 12 enriched NO), mean ± s.e.m. One-tailed unpaired *t*-test, *P = 0.0457 < 0.05. **g**, Schematic of organ bath set-up for contractility analysis. **h**,**i**, Average basal contractility (**h**) and representative traces with quantification (**i**) based on area under the curve (AUC). For **h**, mean ± s.e.m., non-parametric ANOVA (uncorrected Dunn’s test), *P = 0.0148 < 0.05; for **i**, paired non-parametric ANOVA (uncorrected Dunn’s test), ***P = 0.0002, ****P < 0.0001. **j**, Tissue relaxation (ΔAUC) calculated as the difference between NANC and vehicle contractility. Each dot shows a biologically independent colon (*n* = 5 sham, *n* = 13 standard, *n* = 12 enriched NO), mean ± s.e.m. Unpaired ordinary one-way ANOVA with Fisher’s least significant difference test, *P = 0.0475 < 0.05, **P = 0.0029 < 0.01. **k**, Spearman correlation and simple linear regression (two-tailed) between engraftment score and tissue relaxation (as in **j**); two-tailed analysis. Source data

**Extended Data Fig. 9:**
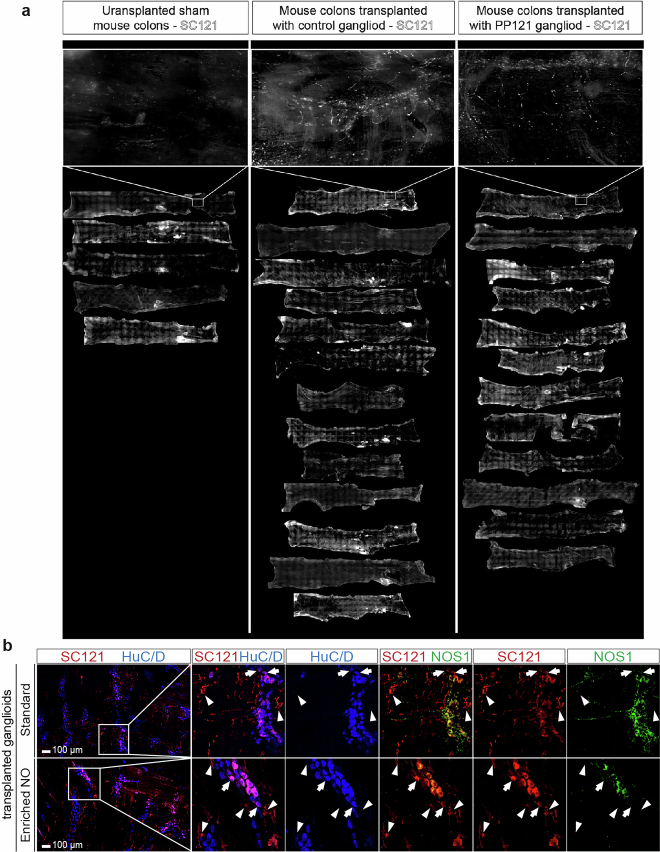
hESC-derived enteric ganglioids engraft in adult mouse colon. **a**) Tiled florescent scanning of whole-mount colonic tissues isolated from sham-operated (left), transplanted colons with standard (middle) or enriched NO neuron (right) ganglioids. One sham-operated animal was lost due to technical issues during sample preparation. **b**) Representative images of IF staining of neuronal marker (HuC/D), SC121 and NOS1 in tissues derived from distal colons of transplanted animals. Arrows point at transplanted non-neuronal (SC121+HuC/D-) cells while arrow heads indicate transplanted non-NO neuronal cells (SC121+HuC/D+NOS1-).

Given the ENS’s limited regenerative capacity, cell transplantation offers a promising strategy to restore neuronal populations in severe enteric neuropathies. Human ENS xenografts also provide a powerful in vivo model to study neuronal circuitry and ENS interactions with the CNS, immune system and microbiome. Previous studies, including ours, have shown successful engraftment of hPSC-derived ENCCs that rescue Hirschsprung disease phenotypes in mice^17^. However, the engraftment ability of fully differentiated hPSC-derived enteric neurons and their potential for treatment of more common adult-onset GI motility disorders have not been explored. It has been shown that the transplanted ex vivo-cultured mouse enteric neurospheres are able to rescue GI motility defects in *Nos1*^*−/−*^ (B6.129S4-Nos1^tm1Plh/^^J^) mice^33^. However, these neurospheres are heterogeneous populations containing only a small percentage of NO neurons. In addition, obtaining sufficient numbers of neurospheres from human primary tissue poses a notable limitation for ultimate regenerative applications. Compared to transplanting ENCC precursors, transplanting mature neurons provides a post-mitotic source of cells with a lower potential clinical risk of tumour formation. To assess the transplantation potential of our enteric ganglioids in *Nos1*^*−/−*^ mice, we injected PP121-treated and untreated enteric ganglioids into the wall of the distal colon in immunocompromised mice and isolated colonic tissue 8 weeks post-surgery for imaging and functional analysis (Fig. 4a). hPSC-derived transplanted cells were detected on the basis of the expression of the human cytoplasmic marker SC121 in the longitudinal muscle myenteric plexus preparations. Notably, we observed a remarkable number of SC121^+^ cells that had spread along the length of the transplanted colons (Fig. 4b, Extended Data Fig. 9a and Supplementary Videos 2–4). Although sham-operated tissues showed only background fluorescence (Fig. 4c), quantifying the SC121^+^ signal revealed that ganglioid-derived cells migrated in transplanted colons, with a higher frequency of SC121^+^ neurons observed closer to the site of transplantation (Fig. 4c). Quantification of the ratio of human NO neurons (NOS1^+^HuC/D^+^SC121^+^) to total human neurons (SC121^+^HuC/D^+^) revealed a higher percentage of NO neurons in colonic tissues transplanted with enriched NO ganglioids (treated with PP121 at day 15–20) compared to tissues transplanted with standard ganglioids (Fig. 4d,e). It is important to note that, although the proportion of NO neurons differed between the transplantation groups, both groups contained SC121^+^HuC/D^−^ non-neuronal human cells as well as SC121^+^HuC/D^+^ human neurons that were negative for NOS1 (Extended Data Fig. 9b).

To investigate whether the engraftment of standard and enriched NO neuron ganglioids could restore nitrergic responses in the *Nos1*^*−/−*^ tissues, we assessed total GI transit time in mice subjected to sham surgery or ganglioid transplants. Remarkably, although transplantation surgery was restricted to the distal region of the colon, a significant reduction in GI transit time was observed in *Nos1*^*−/−*^ animals grafted with enriched NO ganglioids compared to sham-operated *Nos1*^*−/−*^ animals (Fig. 4f). Additionally, there was an observable trend towards improvement in animals transplanted with standard ganglioids (Fig. 4f).

**Extended Data Fig. 10:**
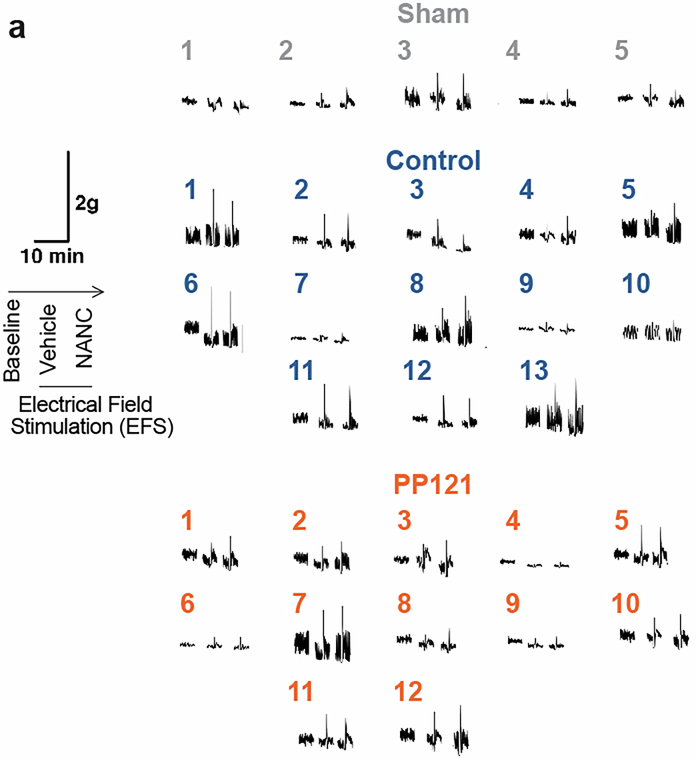
Ex vivo myobath contractility assay traces. **a**) Recorded contractility traces of tension force vs time from mouse distal colonic tissues derived from sham-operated, and transplanted mice.

We prepared longitudinal strips of tunica muscularis near the transplantation site and used our previously described organ bath set-up^33^ to record baseline contractile responses, response to electrical field stimulation (EFS) and EFS-induced responses under non-adrenergic non-cholinergic (NANC) conditions to detect responses mediated by NO (Fig. 4g and Extended Data Fig. 10a). We observed an increased baseline contractility of transplanted tissue sections compared to sham-operated tissues (Fig. 4h). EFS induced a significant relaxation specifically in transplanted colonic tissue (Fig. 4i). Compared to the case for sham-operated, the improvement was more notable if the transplanted ganglioids were enriched in NO (Fig. 4j). The engraftment score (average SC121^+^ expression in distal colon) was correlated with the effect size of NANC-induced relaxation, suggesting that a higher degree of engraftment was associated with stronger relaxation (Fig. 4k).

In addition to the clinically important cell therapy application, the developed human enteric ganglioid xenograft system offers previously unachievable opportunities towards understanding development, physiology and pathophysiology of the human ENS in vivo.

## Discussion

The ENS controls essential GI functions and communicates closely with the brain, immune system and microbiome, playing a central part in gut–brain axis disorders^38–40^. Damage or dysfunction in the ENS, due to genetic factors, environmental stressors or ageing, can lead to debilitating enteric neuropathies that affect millions globally^5,41,42^, yet current treatments are limited to symptom management or surgery.

A major challenge in ENS research is limited access to human ENS tissue owing to its deep location in the gut and the rarity of these cells, which make up only about 1% of the intestinal tissue^18^. Animal models do not fully replicate human ENS pathology; for example, rodents tolerate mutations that cause severe enteric neuropathies in humans^43^. Our hPSC-derived ENS cultures address these limitations by providing scalable, functional sources of human enteric neurons and glia.

The stepwise differentiation system also enabled high-resolution mapping of ENS development, helping uncover mechanisms underlying congenital disorders such as Hirschsprung disease^44,45^. Our models facilitate derivation of disease-relevant subtypes for both mechanistic studies and therapeutic development.

Within the ENS, nitrergic neurons are specifically critical in physiology and disease. Using our 2D ENS cultures, we screened thousands of small molecules to identify factors promoting differentiation into nitrergic neurons. We discovered that PDGFR signalling has a key role in nitrergic fate specification, an insight with translational relevance, given the availability of PDGFR-targeting drugs that may be repurposed for enteric neuropathies.

Our platform identified neuromodulators targeting nitrergic neurons, many of which act on adrenergic, cholinergic and serotonergic receptors and sodium channels. These targets are over-represented in NO neurons, highlighting their specificity. Several compounds approved by the FDA altered colonic motility in ex vivo organ bath assays, marking the identification of candidate drugs that modulate GI motility by targeting nitrergic neurons, laying the groundwork for clinical studies in conditions such as gastroparesis and chronic constipation.

In the past two decades, developing cell-based therapies for enteric neuropathies has been a major area of research^46,47^. In contrast to previous work focusing on ENCC progenitors^17,48^, our study used fully differentiated neurons. We demonstrated successful engraftment of enteric ganglioids into *Nos1*^*–/–*^ mouse colon. Transplanted nitrergic neuron-enriched ganglioids integrated within host ganglia, migrated extensively and rescued GI motility defects. This establishes a path towards cell-based therapies for enteric neuropathies.

Beyond therapy, human ENS xenografts provide a powerful system to study human ENS biology in vivo. These models allow transplantation of patient-derived ganglioids or those bearing specific mutations or stressor exposures, enabling disease modelling and therapeutic testing. Transplanting ganglioids at different differentiation stages supports studies of ENS maturation and fate specification. Furthermore, xenografts provide a tool to investigate ENS crosstalk with gut tissues, the CNS and the microbiome^11,38–40,49,50^.

Enteric neuropathies can arise across the lifespan and remain among the most challenging GI disorders, with causes ranging from congenital mutations to systemic diseases such as diabetes and obesity^5,51,52^. The lack of effective treatments reflects gaps in our understanding of ENS development and function. Our hPSC-derived ENS platforms allow mechanistic and functional studies across diverse genetic and environmental contexts, including patient-specific modelling^53^. Co-cultures with smooth muscle, immune cells and microbiome-derived components could further expand the scope of physiological relevance.

Altogether, our hPSC differentiation strategy enables robust developmental, molecular and functional mapping of human ENS. We offer new insights into NO neuron specification and function, and provide versatile platforms for discovery, modelling and regenerative therapeutics in enteric neurobiology.

## Methods

### Culture and maintenance of undifferentiated human stem cells

The hESC line H9 (WA09-WiCell, RRID:CVCL_9773), along with its reporter-expressing derivatives (hSYN-ChR2–eYFP and NOS1-GFP), and the induced PSC line WTC-11 (UCSFi001-A, RRID:CVCL_Y803), were authenticated and karyotyped by the respective commercial providers. hPSCs were cultured on geltrex-coated plates and maintained in chemically defined medium (E8) as described previously^16^. The cultures were tested for mycoplasma every 30 days.

### ENCC induction

When the monolayer cultures of hPSCs reached about 70% confluency, a previously established 12-day ENCC induction protocol was initiated^16,17^ (day 0) by aspirating the maintenance medium (E8) and replacing it with NC induction medium A (BMP4 (1 ng ml^−1^), SB431542 (10 μM) and CHIR 99021 (600 nM)) in Essential 6 medium). Subsequently, on ENCC induction days 2 and 4, neural crest induction medium B (SB431542 (10 μM) and CHIR 99021 (1.5 μM) in Essential 6 medium), and on days 6, 8 and 10, medium C (medium B with retinoic acid (1 μM)) were fed to the cultures. Next, ENCC crestospheres were formed during day 12–day 15 to facilitate the selection for ENCC lineage and against contaminating ones in our cultures. In doing so, we removed ENCC induction medium C on day 12 and detached the ENCC monolayers using Accutase (30 min, 37 °C, 5% CO_2_). After centrifuging the samples at 290*g* for 1 min, we resuspended the ENCCs in NC-C medium (FGF2 (10 ng ml^−1^), CHIR 99021 (3 μM), N2 supplement (10 μl ml^−1^), B27 supplement (20 μl ml^−1^), glutagro (10 μl ml^−1^) and Minimum Essential Medium non-essential amino acids (MEM NEAAs; 10 μl ml^−1^) in neurobasal medium) and transferred them to ultralow-attachment plates to form free-floating 3D enteric crestospheres. On day 14, when the free-floating enteric crestospheres could be observed, we gently gathered them in the centre of each well using a swirling motion. Then, the old medium was carefully aspirated from the circumference of each well without removing the crestospheres. After addition of the fresh NC-C medium, the cultures were incubated for 24 h (37 °C and 5% CO_2_) before the enteric neuron induction phase.

### Enteric neuron induction from enteric neural crests

On day 15, enteric crestospheres were gathered in the centre of the wells using a swirling motion and NC-C medium was removed using a P1000 micropipette in a slow circular motion, avoiding the free-floating crestospheres. At this step, the protocol varied depending on the final desired culture layout (2D ENS cultures versus 3D enteric ganglioids). For 2D ENS cultures, after washing the enteric crestospheres with PBS, Accutase (Stemcell Technologies, 07920) was added, and plates were incubated for 30 min at 37 °C to dissociate the crestospheres. Then, remaining spheroids were broken by pipetting enteric neuron medium (GDNF (10 ng ml^−1^), ascorbic acid (100 μM), N2 supplement (10 μl ml^−1^), B27 supplement (20 μl ml^−1^), glutagro (10 μl ml^−1^) and MEM NEAAs (10 μl ml^−1^) in neurobasal medium). Cells were spun (2 min, 290*g*, 20–25 °C), and supernatant was removed. Pellet was resuspended in enteric neuron medium, and cells were plated on plates coated with poly-l-ornithine, laminin and fibronectin at 100,000 viable cells per square centimetre. For 3D enteric ganglioids, we avoided Accutase treatment, and enteric crestospheres were fed with the same volume of enteric neuron medium (GDNF (10 ng ml^−1^), ascorbic acid (100 μM), N2 supplement (10 μl ml^−1^), B27 supplement (20 μl ml^−1^), glutagro (10 μl ml^−1^) and MEM NEAAs (10 μl ml^−1^) in neurobasal medium). Feeding continued every other day with enteric neuron medium until day 30–day 40, after which, feeding frequency could be reduced to once or twice per week but with a larger volume of feeding medium.

### Immunofluorescence

For immunofluorescence staining, cells were initially fixed in 4% PFA in PBS (30 min, room temperature) and then blocked and permeabilized by permeabilization buffer (Foxp3/Transcription Factor Staining Buffer Set, 00-5523) for another 30 min at room temperature. After fixation and permeabilization steps, cells were incubated in primary antibody solution overnight at 4 °C, and then washed three times with permeabilization buffer before their incubation with fluorophore-conjugated secondary antibodies at room temperature. Before imaging, stained cells were incubated with DAPI fluorescent nuclear stain and washed an additional three times. The list of antibodies and working dilutions is provided in Supplementary Table 1.

### Preparation of enteric ganglioid frozen sections

hPSC-derived ganglioids were collected, rinsed twice in PBS and fixed on ice in 4% PFA (SCBT sc-281692) for 3 h, followed by replacing 90% of the supernatant with PBS for storage at 4 °C for up to 6 months. Ganglioids were treated with 5% sucrose (RPI Research Products 524060) in PBS for 10 min at room temperature, followed by 10% sucrose in PBS for 2 h at room temperature and 20% sucrose at 4 °C overnight. Sucrose-treated ganglioids were positioned in cryomoulds (Tissue-Tek Cryomold medium, VWR 25608-924), all 20% sucrose was removed and they were incubated in 2:1 20% sucrose/OCT (Tissue Plus O.C.T. Compound Fisher HealthCare 5484) for 2 h at room temperature before flash freezing in ethanol–dry ice. Sections (of 12–20 μm) were taken on a cryostat (Leica 3050S), adhered to a Superfrost Plus Micro Slide, Premium (VWR 48311-703) and dried at 42 °C on a slide dryer for up to 2 h before storing at −80 °C for up to a year.

### Staining enteric ganglioid frozen sections

Unless otherwise specified, all steps were performed at room temperature. Ganglioid frozen sections were prepared as above and then washed three times in PBS and blocked for 1–2 h in serum (10% donkey or 10% goat) with 0.5% (v/v) Triton X-100 (VWR 0694). Slides were then incubated with primary antibody diluted in serum (10% donkey or 10% goat) with 0.1% Triton X-100 at 4 °C for 12–20 h. Slides were washed six times for 20 min each in PBS with 0.1% Tween-20 (Sigma P1379) and incubated for 1 h with Alexa Fluor-conjugated secondary antibodies. The diluted secondary antibody solution was removed and replaced with 1.0 μg ml^−1^ DAPI in water for 10 min. The slides were washed six times for 20 min each in PBS with 0.1% Tween-20, and coverslips were mounted with Fluoromount-G (Southern Biotech 0100-01). The list of antibodies and working dilutions is provided in Supplementary Table 1. Images were acquired on a Leica SP8 inverted confocal or on the Echo Revolve. For images that were stitched, we used Leica’s LAS X tiling feature or the Grid/Pairwise stitching plugin for FIJI^54^.

### Two-photon fluorescence imaging

Imaging experiments were conducted on a custom-built upright two-photon microscope operating with µManager software (San Francisco, CA). The excitation source was a two-photon Coherent Chameleon Vision II laser operating at 760 nm. Images were collected using an Olympus LWD 1.05-numerical-aperture water immersion objective (Olympus). An emission filter collecting light between 380 nm and 420nm (Chroma) was used to image DAPI, whereas the fluorescence emission of Alexa 568 was collected using a filter between 565 nm and 635 nm (Chroma).

### Macro fluorescence imaging

Images were taken on a Nikon AZ100M ‘Macro’ laser scanning confocal instrument configured with long-working-distance low-magnification lenses. The microscope is equipped with the standard 405 nm, 488 nm, 561 nm and 640 nm laser lines and has photomultiplier tube detectors with a detection range from 400 nm to 700 nm. To reduce signal drop-off at the image edges, we used an optical zoom factor of ×2.1 and increased our lateral resolution using a digital zoom factor of ×1.873.

### Flow cytometry

For preparation of samples for flow cytometry analysis, cells were initially dissociated into single-cell suspensions by Accutase treatment (Stemcell Technologies, 07920, 30–60 min, 37 °C, 5% CO_2_) and then fixed and permeabilized using fixation and permeabilization buffers (Foxp3/Transcription Factor Staining Buffer Set, 00-5523). Cells were stained with primary and secondary antibodies as described above for immunofluorescence. Flow cytometry was conducted using a BD LSRFortessa cell analyser and data were analysed using Flowjo (Software Version *8.7*). The list of antibodies and working dilutions is provided in Supplementary Table 1.

### hSYN-ChR2–eYFP enteric ganglioid blue-light activation

Enteric ganglioids were either exposed to blue light (100% laser intensity, 3 × 1-min exposure with 30-s intervals, EVOS FL) or left out in ambient light. Enteric ganglioids were then incubated for 45 min at 37 °C before dissociation, fixation and permeabilization for flow cytometry (see above). Cells were stained using antibodies to cFOS (Abcam, ab190289) and TUBB3 (Biolegend, 801202).

### Bulk RNA-sequencing data analysis

Total RNA was extracted using the PureLink RNA Mini Kit. First-strand cDNA was then synthesized with the Quantseq Forward Library preparation kit from Lexogen. Illumina-compatible RNA-sequencing libraries were prepared with Quantseq and pooled and sequenced on an Illumina Hiseq 4000 platform at the University of California, San Francisco (UCSF) Center for Advanced Technology. Unique molecular identifiers were extracted from the fastq files with umi_tools, and cutadapt was used to remove short and low-quality reads. The reads were aligned to the human GENCODE v.34 reference genome using STAR aligner, and the duplicate reads were collapsed using umi_tools. Gene level counts were measured using HTSeq and compared using DESeq2.

### Single-cell and single-nucleus RNA-sequencing sample preparation and data collection

All tubes and pipette tips used for cell collection were pretreated with 1% BSA in 1× PBS. Cells were dissociated in Accutase (Stem Cell) at 37 °C, in 10-min increments, with end-to-end rotation, until a single-cell suspension was obtained. The cells were washed in Cell Staining Buffer (Biolegend) and stained with TotalSeq HTO antibodies for 30 min on ice. The cells were washed twice in Cell Staining Buffer and filtered through a 40-µm pipette tip strainer (BelArt). The cells were counted using Trypan blue dye and a haemocytometer and pooled for sequencing. Single-cell RNA-sequencing libraries were prepared with Chromium Next GEM Single Cell 3′ Kit v3.1 (10x Genomics), with custom amplification of TotalSeq HTO sequences (Biolegend). The libraries were sequenced on an Illumina NovaSeq sequencer in the Center for Advanced Technologies (UCSF). The cell feature matrices were extracted using kallisto/bustools, and demultiplexed using seurat.

### Quality control and cell filtration

Datasets were analysed in R v4.0.3 with Seurat v4 (ref. ^55^). The number of reads mapping to mitochondrial and ribosomal gene transcripts per cell was calculated using the PercentageFeatureSet function. Cells were identified as poor quality and subsequently removed independently for each dataset on the basis of the number of unique features captured per cell, the number of unique molecular identifiers captured per cell and the percentage of reads mapping to mitochondrial transcripts per cell. Dataset-specific quality control metric cutoffs can be found in Supplementary Table 2.

### Dimensionality reduction, clustering and annotation

In cases in which it was applicable, biological replicate samples were first merged using the base R merge function. Count matrices were log-normalized with a scaling factor of 10,000, and 2,000 variable features were identified using the vst method. For datasets specified in Supplementary Table 3, count matrices of biological replicate samples were integrated using Seurat integration functions with default parameters. Cell cycle phase was predicted using the CellCycleScoring function with Seurat’s S and G2M features provided in cc.genes. The variable feature sets were scaled and centred, and the following variables were regressed out: nFeatures, nCounts, mitochondrial gene percentage, ribosomal gene percentage, S score and G2M score. Principal component analysis was run using default settings, and UMAP dimensionality reduction was performed using the principal component analysis reduction. The shared nearest neighbour (SNN) graph was computed using default settings, and cell clustering was performed using the default Louvain algorithm. Quality control metrics were visualized per cluster to identify and remove clusters of low-quality cells (less than average nFeatures or nCounts and higher than average mitochondrial and ribosomal gene percentage; Supplementary Table 3). The above pipeline was performed again on datasets after the removal of any low-quality cell clusters and for the subclustering analysis of the enteric neural crest, enteric neurons, nitrergic neurons and enteric glia. The number of principal components used for UMAP reduction and SNN calculation was determined by principal component standard deviation and varied for each dataset. The number of principal components used for SNN and UMAP calculation and the resolution used for clustering of each dataset are available in Supplementary Table 3. Cluster markers were found using the Wilcoxon rank sum test, and clusters were annotated on the basis of the expression of known cell-type marker genes (Supplementary Table 4). Following cell-type annotation, gene dropout values were imputed using adaptively thresholded low-rank approximation (ALRA)^56^. The rank-*k* approximation was automatically chosen for each dataset, and all other parameters were set as the default values. The imputed gene expression is shown in all plots and used in all downstream analysis unless otherwise specified.

### Analysis of published datasets

#### Quality control

Criteria used by the original authors of each dataset were used to identify and remove poor-quality cells. Dataset-specific quality control metric cutoffs are provided in Supplementary Table 2.

#### Dimensionality reduction and clustering

Datasets were analysed with Seurat using the methods and parameters described by the original authors.

#### AR-human

For all datasets, count matrices were log-normalized with a scaling factor of 10,000, and 2,000 variable features were identified using the vst method. Batch correction by Unique_ID was performed using mutual nearest neighbours correction with the RunFastMNN Seurat Wrappers function. The dataset-specific parameters used for the RunUMAP, FindNeighbors and FindClusters functions are provided in Supplementary Table 3. Cell annotations determined by the authors were used for cell types and neuronal subtypes.

#### ST-human

Datasets downloaded from the Gut Cell Atlas (https://www.gutcellatlas.org/) retained the author’s original clustering annotations and dimensionality reduction coordinates.

For consistency of comparison, gene dropout values were imputed using adaptively thresholded low-rank approximation for all published datasets using automatically determined rank-*k* approximations and all other default values. The imputed gene expression is shown in all plots and used in all downstream analysis unless otherwise specified.

### Cell-type transcriptional signature scoring

To find transcriptionally similar cell populations between two datasets, first the differentially expressed genes of the reference dataset were calculated from the non-imputed gene counts with the FindAllMarkers function using the Wilcoxon rank sum test, and only genes with a positive fold change were returned. The differentially expressed gene lists were first filtered to remove genes not present in the query dataset. Then for each cell cluster in the reference dataset, a transcriptional signature gene list was made from the top 100 differentially expressed genes sorted by increasing adjusted *P* value. The query dataset was then scored for the transcriptional signature gene lists of each reference dataset cell cluster using the AddModuleScore function on the basis of the query dataset’s imputed gene counts.

To identify nitrergic neurons, enteric neurons that expressed NOS1^+^ were scored for a module consisting of the rate-limiting synthesis enzyme(s), metabolism enzymes and transport proteins (NOS1, NOS1AP, ARG1, ARG2, ASL and ASS1) using the AddModuleScore function. A neuron was then annotated as nitrergic or NO if the cell’s expression of NOS1 was greater than 0 and the cell’s module score for the module above was greater than 0. A cell was annotated as other if both criteria were not met.

### PP121 versus control gene expression correlation

To compare the gene expression of control and PP121-treated cell types, and neuronal subtypes and NO neuron subtypes, a subset dataset of each cell-type and subtype annotation was first created. For each subset, the non-imputed average expression of all genes was then calculated for the control and PP121-treated cells using the AverageExpression function and natural log-transformed for plotting. *R*^2^ values comparing the control and PP121 natural log expression values were calculated from linear modelling using the *y* ~ *x* formula, where *y* is modelled as a function of *x*.

### cFOS expression screening

Day 90 enteric ganglioids were dissociated using Accutase, and single-cell suspensions (in ENC medium) were distributed in wells of V-bottom 96-well plates. Compounds from a neuronal signalling compound library (Selleckchem) were added at 1 μM using a pin tool, and cells were incubated for 75 min at 37 °C. Afterwards, cells were washed with PBS, and were immediately fixed for flow cytometry.

### NO release assay

For high-throughput measures of NO release, day-40 2D ENS cultures (96-well plates) were used. After washing cells with Tyrode’s solution (NaCl (129 mM), KCl (5 mM), CaCl_2_ (2 mM), MgCl (1 mM), glucose (30 mM) and HEPES (25 mM) at pH 7.4), 70 μl of Tyrode’s solution was added to each well. Neuronal signalling compounds (Selleckchem) were added at 1 μM using a pin tool. After a 45-min incubation at 37 °C, supernatants were used to determine NO release using an NO assay kit (Invitrogen, EMSNO). In brief, the kit uses the enzyme nitrate reductase, which converts nitrate to nitrite that is then detected as a coloured azo dye absorbing light at 540 nm. NO release for each compound was presented as the *A*_540nm_ relative to the vehicle (dimethylsulphoxide).

### High-throughput screening to identify compounds that enrich NO neurons

Day-15 enteric crestospheres derived from H9 hESCs were dissociated into single cells (Accutase, Stemcell Technologies, 07920, 30 min, 37 °C), resuspended in enteric neuron medium and transferred into 384-well plates. Plates were incubated for 2 h for cells to attach. Using a pin tool, drugs from a library of 1,694 inhibitors (SelleckChem) were added to wells at the final concentration of 1 μM, and plates were incubated with drugs until day 20, when medium was changed to enteric neuron medium with no drugs. At day 40, cells were fixed, stained for NOS1 and imaged using an InCellAnalyzer 2000 (GE Healthcare). Hits were selected on the basis of the fold increase of the percentage of NOS1^+^ cells relative to the wells treated with vehicle (dimethylsulphoxide).

### Drug–target interaction prediction

We obtained canonical SMILES of our hits from PubChem^4,5^) and generated a list of their known and predicted targets by combining data from the following databases: BindingDB (https://www.bindingdb.org/), Carlsbad (http://carlsbad.health.unm.edu/), DINIES (https://www.genome.jp/tools/dinies/), PubChem BioAssay (https://pubchem.ncbi.nlm.nih.gov/, filtered for active interactions), SEA (http://sea.bkslab.org/, filtered for MaxTC >0.4), SuperDRUG2 (http://cheminfo.charite.de/superdrug2/) and SwissTargetPrediction (http://www.swisstargetprediction.ch/).

### In vivo cell transplantation

Specific pathogen-free homozygote neuronal nitric oxide synthase-knockout mice (B6.129S4-Nos1^tm1Plh^/J; *nNos1*^*−/−*^) were bred and maintained, in individually ventilated cages, for use as recipients. Animals used for these studies were maintained, and the experiments were performed, in accordance with the UK Animals (Scientific Procedures) Act 1986 and approved by the University College London (UCL) Biological Services Ethical Review Process. Animal husbandry at UCL Biological Services was practised in accordance with the UK Home Office Certificate of Designation. This was practised according to the institute’s standard protocols, and no impact on the experimental outcomes was anticipated. As *Nos1*^*−/−*^ mice are immunocompetent, cyclosporin A (250 μg ml^−1^ in drinking water) was administered orally 2 days before transplantation to reduce the possible rejection of donor human cells. Cyclosporin A-treated *Nos1*^*−/−*^ mice from both sexes were chosen at random, from within littermate groups, and day-40–60 enteric ganglioids were transplanted to the wall of the distal colon of postnatal day 23–27 mice, through laparotomy under isoflurane anaesthetic. In brief, the distal colon was exposed and enteric ganglioids, containing 0.5–1 million cells, were subsequently transplanted to the serosal surface of the distal colon, by mouth pipette, using a pulled glass micropipette. Each transplanted tissue typically received three ganglioids, which were manipulated on the surface of the distal colon, with the bevel of a 30-G needle, to ensure appropriate positioning. Transplanted *Nos1*^*−/−*^ mice were maintained with continued free access to cyclosporin A (250 μg ml^−1^)-treated drinking water for up to 8 weeks post-transplantation, to ensure extended immunosuppression, before euthanization and removal of the colon for analysis. The sample size for this study was determined on the basis of previous experience and known variability in these transplantation and engraftment assays. No statistical test was performed to determine the sample size. Cages and animals were randomly assigned to experimental groups to reduce potential bias. Blinding was not performed during transplantation or analysis. As cyclosporin A can affect several signalling pathways and induce gene expression changes, it is crucial to verify immunofluorescence results using appropriate controls such as tissue from cyclosporin A-treated untransplanted animals in follow-up studies. In addition, other immunocompromised backgrounds (for example, NSG) will be important to further verify these engraftment results.

### Tissue preparation and fixation

Following the excision, the entire colon was pinned in a Sylgard (Dow)-lined Petri dish and opened along the mesenteric border. The mucosa was subsequently removed by sharp dissection, and tissues were fixed in 4% PFA in PBS (45 min–1 h, 22 °C) for further processing.

### Tissue staining

Colonic longitudinal muscle myenteric plexus tissues were fixed with 4% PFA (1 h on ice), Thermo Scientific, J19943-K2) and blocked and permeabilized with a buffer containing 1% BSA and 1% Triton X-100 (in PBS, 45 min, room temperature). Then, tissues were incubated with primary antibody solutions (in the same buffer, overnight, 4 °C) and were washed three times before treatment with fluorophore-conjugated secondary antibodies (1 h, room temperature). Samples were stained with DAPI and washed before mounting using Vectashield (Vector Laboratories, H-1400). Antibodies are listed in Supplementary Table 1.

### MEA analysis

#### Data acquisition

Neuron activity was recorded with the Axion Maestro Edge on Cytoview MEA 24-well plates in 1-h recording sessions for each condition. Neuormodulator or vehicle were added by removing the plate from the Maestro Edge, half-changing the medium with 2× concentrated neuromodulator or vehicle in pre-warmed medium, and immediately returning the plate to the Axion to resume recording. Optogenetic stimulation was performed with the Axion Lumos attachment, stimulating all wells of the plate with 488-nm light at 50% intensity, 1 s on, 4 s off, 30 times.

#### Data processing

Raw data were first spike-sorted with a modified version of SpikeInterface (https://github.com/SpikeInterface) using MountainSort to identify high-quality units by manually scoring on the basis of amplitude, waveform shape, firing rate and inter-spike interval contamination. For pharmacology experiments, neurons were matched between vehicle and neuromodulator recordings by examining all detected units on a specific electrode after spike scoring and identifying units with identical waveforms. Firing rates of these ‘paired’ units from all wells that received the treatment were compared across the control and neuromodulator conditions. Positive responders were units that had a firing rate change greater than +0.1 Hz; negative responders had a firing rate change less than −0.1 Hz; neutral responders had a firing rate change between −0.1 and +0.1 Hz. For optogenetic experiments, individual units were again extracted with SpikeInterface and manually scored. Recordings were separated into ‘on’ times when the LED was active and ‘off’ times when it was not. All units were compiled, and firing rates for each unit were compared during the on and off windows.

### Calcium imaging

Day-5 enteric ganglioids were plated on plates coated with poly-l-ornithine, fibronectin and laminin to be compatible with a microscopy-based calcium imaging assay and cultured until day 50. For real-time calcium imaging, medium was removed, and cultures were washed once with Tyrode’s solution (Boston Bioproducts, BSS-355). Then cultures were incubated with Tyrode’s solution supplemented with 2.5 μg ml^−1^ Fluo-4 AM (1041F, Ion Biosciences) and 1:100 Pluronic F-127 (Ion Biosciences) for 30 min at 37 °C. Then, dye-containing solution was completely removed and replaced with pre-warmed Tyrode’s solution. Plates were transferred to an Echo Revolve microscope, and baseline videos were recorded. During recording, Tyrode’s buffer containing 2× concentrated neurotransmitters (for a final concentration of 100 M) was added and recording was continued for an extra 30 s. Snapshots before and after neurotransmitter addition were used for quantification using FIJI^57^. For analysis, fluorescence intensities were quantified in the same regions of interest defined using automatically identified particles using a globally applied threshold. Fluorescent intensity calculations included all pixels within the region of interest. The data were reported as normalized fluorescence intensity for each particle, calculated by dividing the intensity after addition by the baseline intensity for each particle.

### Ex vivo whole-organ colonic motility assays

#### Preparation of solutions

Krebs buffer (NaCl (117 mM), KCl (4.7 mM), NaH_2_PO_4_ (1.2 mM), MgCl_2_ (1.5 mM), CaCl_2_.2H_2_O (2.5 mM), NaHCO_3_ (25 mM), glucose (11 mM), pH 7.4) was placed in a 37 °C water bath and aerated with 95% O_2_ and 5% CO_2_ (carbogen) gas mixture for at least 30 min before experiment onset. ‘Drug’ treatment solutions were freshly prepared by adding the drug compound into Krebs buffer before starting data acquisition. The solution with NOS1 inhibitor was prepared by adding l-NAME to the drug solutions making drug + l-NAME.

#### Tissue dissection

For each experimental replicate, a pair of 8-week-old wild-type C57BL6 mice (male) were placed in a sealed chamber and euthanized using CO_2_ asphyxiation followed by cervical dislocation. The lower GI tract (caecum and colon) was removed and immediately transferred to 37 °C carbogenated Krebs buffer, with the faecal matter still inside. Adipose tissue and mesentery were removed before placing the colons in the organ bath reservoir of GI motility monitor (GIMM) apparatus. GIMM had two reservoirs making simultaneous acquisition of control and drug-treated colons possible.

#### Experimental set-up and procedure

GIMM was designed on the basis of a previously reported model^58^. The organ reservoir of GIMM has two chambers for recording two specimens simultaneously. It is connected to working solutions kept at 37 °C via a four-channel peristaltic pump (WPI, PERIPRO-4LS). Lower GI tract was collected and transferred to the organ bath with Krebs buffer flowing through. The caecum was pinned down at the proximal tip, and the distal end of the colon was pinned through the serosa and/or mesentery. Five 10-min (for the initial drug testing) or sequential 6-min (for the sequential drug treatment in the presence and absence of l-NAME) videos were recorded using the IC capture software (Imaging Source) with a high-resolution monochromatic firewire industrial camera (Imaging Source, DMK41AF02) connected to a 16-mm *f*/1.4 C-Mount Fixed Focal Lens (Fujinon HF16SA1). Tissue in the control chamber was exposed only to Krebs solution; the order of solutions in the experimental chamber was: Krebs, drug compound, Krebs (6 min each), l-NAME (2 min), l-NAME in the presence of a drug compound (6 min) and Krebs (6 min). The chambers were cleaned after each acquisition. Cages and animals were randomly assigned to experimental groups to reduce potential bias. Investigators were blinded during data acquisition but not blinded during data analysis.

#### Data and statistical analysis

VolumetryG9a was used to generate the spatiotemporal map of each acquisition^59^. Slow-wave and CMMC data were generated from spatiotemporal maps. Statistical analyses were performed using PRISM. The Kolmogorov–Smirnov test was used to compare cumulative frequency distributions in the CMMC and slow-wave analyses. For LCE count analysis, we performed one-way ANOVA.

### GI transit analysis in mice

Mice were fasted for a period of 1 h before administration of 100 μl Brilliant Blue FCT (E133) solution (Cambridge Bioscience) to the stomach, by gavage, at 8 weeks post-transplantation. Following gavage, mice were returned to individual cages, with free access to food and water, and monitored continuously for visualization of dye in the stool. Total GI transit time was calculated from time of administration to the first visualization of dye in the stool (minutes).

### Contractility analysis of colonic tissue

Longitudinal colonic muscle strips were isolated, and the mucosa was removed by sharp dissection in oxygenated Krebs solution. Longitudinal strips of the tunica muscularis were mounted in tissue baths (10 ml, SI-MB4; World Precision Instruments) connected to force transducers (SI-KG20, World Precision Instruments), via suture, under an initial tension of 0.5 g. Tissues were maintained at 37 °C with perfusion of oxygenated Krebs solution. Following a 60-min equilibration period, activity was recorded using a Lab-Trax-4 data acquisition system (World Precision Instruments) in the absence and presence of NANC conditions (atropine, 1 μM; phentolamine hydrochloride, 1 μM; propranolol hydrochloride, 1 μM). Following a basal recording of 20–25 min, EFS was applied for 30 s as trains of electrical pulses (5 Hz; 40 V; 0.3-ms pulse duration) at 5-min intervals, via platinum electrode loops placed at each end of the muscle strip, using a MultiStim System (D330, World Precision Instruments). Three rounds of EFS were applied in each condition to ensure that reproducible responses were observed. The total recording time for individual muscle strips was 1 h 45 min. Data were collected, stored and analysed by computer using a data acquisition program (Labscribe V4, World Precision Instruments).

### Generating figure schematics

We used Adobe Illustrator (version 25.4.1) to generate schematics for the figures. Microsoft Excel version 16.96.1 and Graphpod Prism version 10 were used to generate graphs, and statistical data analysis. R v4.0.3 with Seurat v4 was used for single-nucleus RNA-sequencing data analysis.

### Use of hESCs

This study used established and commercially available hESC lines and was approved by the UCSF Human Gamete, Embryo, and Stem Cell Research Committee.

### Ethical approval statement for the animal study

Animals used for these studies were maintained, and the experiments were performed, in accordance with the UK Animals (Scientific Procedures) Act 1986 and approved by the UCL Biological Services Ethical Review Process. Animal husbandry at UCL Biological Services was in accordance with the UK Home Office Certificate of Designation. All procedures followed the National Institutes of Health Guidelines for the Care and Use of Laboratory Animals and were approved by the Stanford University Administrative Panel on Laboratory Animal Care.

### Reporting summary

Further information on research design is available in the Nature Portfolio Reporting Summary linked to this article.

## Online content

Any methods, additional references, Nature Portfolio reporting summaries, source data, extended data, supplementary information, acknowledgements, peer review information; details of author contributions and competing interests; and statements of data and code availability are available at 10.1038/s41586-025-09208-3.

## Supplementary information

Supplementary Tables Supplementary Tables 1–4.

Reporting Summary

Supplementary Video 1 Representative video of the effect of dexmedetomidine on mouse colonic motility ex vivo.

Supplementary Video 2 Representative video of SC121^+^ human cell engraftment into the oral region of a *Nos1*^*−/−*^ mouse colon 8 weeks post-surgery.

Supplementary Video 3 Representative video of SC121^+^ human cell engraftment into the middle region of a *Nos1*^*−/−*^ mouse colon 8 weeks post-surgery.

Supplementary Video 4 Representative video of SC121^+^ human cell engraftment into the aboral region of a *Nos1*^*−/−*^ mouse colon 8 weeks post-surgery.

## Source data

Source Data Fig. 2

Source Data Fig. 4

Source Data Extended Data Fig. 5

Source Data Extended Data Fig. 6

Source Data Extended Data Fig. 7

## Data Availability

Data supporting the results of this manuscript are available within the article, its figures and Supplementary Information. The raw and processed datasets from single-nucleus RNA sequencing of hESC-derived ENS cultures are available via the Gene Expression Omnibus under the accession number GSE196592. Source data are provided with this paper.

## Code availability

This paper does not report original code.
