# Supplementary material for: Engrafted nitrergic neurons derived from hPSCs improve gut dysmotility in mice
Source: Nature. 2025 Jun 25;645(8079):158–67. doi: 10.1038/s41586-025-09208-3 (PMC12408359; doi:10.1038/s41586-025-09208-3)
Supplement: Supplementary file 1 — Supplementary Tables 1–4. [file 41586_2025_9208_MOESM1_ESM.pdf]

nature portfolio

https://doi.org/10.1038/s41586-025-09208-3

Supplementary information

Engrafted nitrergic neurons derived from hPSCs improve gut dysmotility in mice

In the format provided by the authors and unedited

Table S1 Antibodies

Antibody	Host	Vendor	cat number	used for	dilution
cFOS	rabbit	Abcam	ab190289	IF, FC	1:2500, 1:2500
CHAT	rabbit	Proteintech	20747-1-AP	IF	1:1000
GABA	rabbit	Sigma	a2052	IF, FC	1:2500
GFAP	chicken	abcam	ab4674	IF	1:2500
HuC/D	mouse	invitrogen	a21271	IF	1:250
NOS1	mouse	Santa Cruz Biotechnology	sc-5302	IF, FC	1:50 (IF), 1:100
NOS1	rabbit	Invitrogen	61-7000	FC	1:200
PDGFRA	rabbit	Cell Signaling	5241	FC	1:100
PDGFRB	rat	eBioscience	14-1402-82	FC	1:500
PMP22	rabbit	abcam	ab203053	IF	1:100
S100	rabbit	Thermo Scientific	RB-9018-P0	IF	1:500
Serotonin	rabbit	Sigma	s5545	IF, FC	1:8000
STEM121	mouse	Takara Bio	Y40410	IF	1:1000
TUBB3	chicken	Millipore Sigma	ab9354	FC	1:350
TUBB3	mouse IgG2a	Biolegend	801202	IF	1:1500
anti chicken IgY (H+L) Alexa Fluor 488	donkey	Invitrogen	A78948	IF, FC	1:1000
anti chicken IgY (H+L) Alexa Fluor 647	donkey	Invitrogen	A78952	IF, FC	1:1000
anti mouse IgY (H+L) Alexa Fluor 488	donkey	Invitrogen	A21202	IF, FC	1:1000
anti mouse IgY (H+L) Alexa Fluor 568	donkey	Invitrogen	A10037	IF, FC	1:1000
anti mouse IgY (H+L) Alexa Fluor 647	donkey	Invitrogen	A31571	IF, FC	1:1000
anti mouse IgG1a Alexa Fluor 568	goat	Invitrogen	A21124	IF, FC	1:1000
anti mouse IgG1a Alexa Fluor 647	goat	Invitrogen	A21240	IF, FC	1:1000
anti mouse IgG2a Alexa Fluor 488	goat	Invitrogen	A21131	IF, FC	1:1000
anti mouse IgG2b Alexa Fluor 568	goat	Invitrogen	A21144	IF, FC	1:1000
anti rabbit IgY (H+L) Alexa Fluor 647	donkey	Invitrogen	A31573	IF, FC	1:1000
anti rat IgY (H+L) Alexa Fluor 647	donkey	abcam	ab150155	IF, FC	1:1000

Table S2 QC Metrics

Dataset	nFeatures greater than	nFeatures less than	nCounts less than	percent mitochondrial reads less than
D40 ganglioid control BR1	1250	7000	40000	10%
D40 ganglioid control BR2	1250	7000	40000	10%
D40 ganglioid pp121 BR1	1250	7000	40000	10%
D40 ganglioid pp121 BR2	1250	7000	40000	10%
D10	1250	3500	20000	5%
D15	1500	3500	20000	10%
Drokhlyansky et al.				
Human	1000	NA	NA	NA

Table 3 Clustering parameters

Dataset	Cell Type Subset	Batch Correction Method	# PCs Used (SNN and UMAP)	UMAP min.dist	UMAP n.neighbors	UMAP metric	UMAP umap.method	Clustering Resolution	Cluster(s) removed and re-clustered? (Reason)
D40 ganglioid Control	All Cells	NA (regular merge)	18	0.3	30	cosine	uwot	0.4	Yes (High ribosomal gene percentage)
D40 ganglioid Control	Neurons	NA (regular merge)	17	0.3	30	cosine	uwot	0.6	Yes (High ribosomal gene percentage)
D40 ganglioid Control	Nitrergic Neurons	NA (regular merge)	14	0.3	30	cosine	uwot	0.3	
D40 ganglioid Control and PP121	All Cells	NA (regular merge)	18	0.3	30	cosine	uwot	0.2	
D40 ganglioid Control and PP121	Neurons	NA (regular merge)	16	0.3	30	cosine	uwot	0.6	Yes (High ribosomal gene percentage)
D40 ganglioid Control and PP121	Nitrergic Neurons	NA (regular merge)	16	0.3	30	cosine	uwot	0.3	
D10	All Cells	NA	11	0.3	30	cosine	uwot	0.1	
D10	ENCs	NA	17	0.3	30	cosine	uwot	0.2	Yes (High expression of placode genes)
D15	All Cells	NA	16	0.3	30	cosine	uwot	0.1	
D15	ENCs	NA	21	0.3	30	cosine	uwot	0.5	Yes (High SYP expression)
Drokhlyansky et al.									
Human	All Cells	MNN	30	0.3	30	cosine	uwot	0.1	
Human	Neurons	MNN	30	0.3	30	cosine	uwot	0.1	
Human	Nitrergic Neurons	MNN	30	0.3	30	cosine	uwot	0.6	

Table S4 Cell Type Annotation Genes

Enteric Neurons	Progenitor	Epithelial	Mesenchymal
NRXN3	NOTCH1	CDH1	PRRX1
NRXN1	SOX2	EPCAM	RUNX2
DCX	MEF2C	KRT19	TWIST1
MAPT	PAX3		COL11A1
ELAVL2	PAX7		COL1A2
NRCAM	NFIA		COL1A1
RBFOX3	MKI67		COL3A1
NCAM1			COL5A2
NRG1			FN1
SYN1			LAMA4
SYP			EDNRA
			PDGFRA
			PDGFRB
