# Supplementary material for: Engrafted nitrergic neurons derived from hPSCs improve gut dysmotility in mice
Source: Nature. 2025 Jun 25;645(8079):158–67. doi: 10.1038/s41586-025-09208-3 (PMC12408359; doi:10.1038/s41586-025-09208-3)
Supplement: Supplementary file 2 — Reporting Summary [file 41586_2025_9208_MOESM2_ESM.pdf]

nature portfolio

Corresponding author(s): Faranak Fattahi, PhD

Last updated by author(s): May 27, 2025

Reporting Summary

Nature Portfolio wishes to improve the reproducibility of the work that we publish. This form provides structure for consistency and transparency in reporting. For further information on Nature Portfolio policies, see our Editorial Policies and the Editorial Policy Checklist.

Statistics

For all statistical analyses, confirm that the following items are present in the figure legend, table legend, main text, or Methods section.

n/a	Confirmed	
☐	☒	The exact sample size (*n*) for each experimental group/condition, given as a discrete number and unit of measurement
☐	☒	A statement on whether measurements were taken from distinct samples or whether the same sample was measured repeatedly
☐	☒	The statistical test(s) used AND whether they are one- or two-sided<br>*Only common tests should be described solely by name; describe more complex techniques in the Methods section.*
☒	☐	A description of all covariates tested
☐	☒	A description of any assumptions or corrections, such as tests of normality and adjustment for multiple comparisons
☐	☒	A full description of the statistical parameters including central tendency (e.g. means) or other basic estimates (e.g. regression coefficient) AND variation (e.g. standard deviation) or associated estimates of uncertainty (e.g. confidence intervals)
☐	☒	For null hypothesis testing, the test statistic (e.g. *F*, *t*, *r*) with confidence intervals, effect sizes, degrees of freedom and *P* value noted<br>*Give P values as exact values whenever suitable.*
☒	☐	For Bayesian analysis, information on the choice of priors and Markov chain Monte Carlo settings
☐	☒	For hierarchical and complex designs, identification of the appropriate level for tests and full reporting of outcomes
☐	☒	Estimates of effect sizes (e.g. Cohen's *d*, Pearson's *r*), indicating how they were calculated

Our web collection on statistics for biologists contains articles on many of the points above.

Software and code

Policy information about availability of computer code

Data collection	scRNA-seq libraries were prepared with Chromium Next GEM Single Cell 3′ Kit v3.1 (10x Genomics), with custom amplification of TotalSeq HTO sequences (Biolegend). The libraries were sequenced on Illumina NovaSeq sequencer in the Center for Advanced Technologies (UCSF). The cell feature matrices were extracted using kallisto/bustools, and demultiplexed using Seurat. We used the following available software, computational packages and packages: R v4.0.3 with Seurat v4,
Data analysis	We used the following open access codes and packages: R v4.0.3 with Seurat v4. Adobe Illustrator, Microsoft Excel (version 16.96.1) and Graphpod Prism (version 10) for generating schematics, graphs, and statistical data analysis. For GITT ex vivo analysis we used Volumetry G9a (Spear et al 2018).

For manuscripts utilizing custom algorithms or software that are central to the research but not yet described in published literature, software must be made available to editors and reviewers. We strongly encourage code deposition in a community repository (e.g. GitHub). See the Nature Portfolio guidelines for submitting code & software for further information.

Data

Policy information about availability of data

All manuscripts must include a data availability statement. This statement should provide the following information, where applicable:

- Accession codes, unique identifiers, or web links for publicly available datasets
- A description of any restrictions on data availability
- For clinical datasets or third party data, please ensure that the statement adheres to our policy

The raw and processed datasets from bulk and scRNA-seq of hESC-derived cells are available as of the date of publication on GEO under accession number (GSE196592). No original code was generated for this paper. Data supporting the results of this manuscript are available within the article, its figures and supplementary material. The raw and processed datasets from sequencing of hESC-derived ENS cultures are available under accession number GEO: GSE196592.

Research involving human participants, their data, or biological material

Policy information about studies with human participants or human data. See also policy information about sex, gender (identity/presentation), and sexual orientation and race, ethnicity and racism.

Reporting on sex and gender	N/A
Reporting on race, ethnicity, or other socially relevant groupings	N/A
Population characteristics	N/A
Recruitment	N/A
Ethics oversight	N/A

Note that full information on the approval of the study protocol must also be provided in the manuscript.

Field-specific reporting

Please select the one below that is the best fit for your research. If you are not sure, read the appropriate sections before making your selection.

☒ Life sciences ☐ Behavioural & social sciences ☐ Ecological, evolutionary & environmental sciences

For a reference copy of the document with all sections, see nature.com/documents/nr-reporting-summary-flat.pdf

Life sciences study design

All studies must disclose on these points even when the disclosure is negative.

Sample size	The sample size for this study was determined based on the primary outcome measure. This calculation assumed a standard deviation based on previous studies. To account for potential dropouts and missing data, we increased the sample size. Except for high-throughput drug screenings (n=1), at least n=3 were used for the reported data. High-throughput hits were confirmed and validated further.
Data exclusions	A transplanted animal was excluded from the gastrointestinal analysis due to technical error and difficulty reading the baseline recordings in ex vivo set up. This was as a result of tissue viability and quality.
Replication	The reproducibility of the presented data were confirmed using independent samples (cell differentiations, samples, animals) at least with n=3 and higher as specified in the text.
Randomization	Sample allocations were random.
Blinding	Investigators were blinded to group allocations. In other cases, (drug treatments), same samples were equally distributed between treatment groups and were paired. Such data are explained clearly in the text.

Reporting for specific materials, systems and methods

We require information from authors about some types of materials, experimental systems and methods used in many studies. Here, indicate whether each material, system or method listed is relevant to your study. If you are not sure if a list item applies to your research, read the appropriate section before selecting a response.

Materials & experimental systems

n/a	Involved in the study	
☐	☒	Antibodies
☐	☒	Eukaryotic cell lines
☒	☐	Palaeontology and archaeology
☐	☒	Animals and other organisms
☒	☐	Clinical data
☒	☐	Dual use research of concern
☒	☐	Plants

Methods

n/a	Involved in the study	
☒	☐	ChIP-seq
☐	☒	Flow cytometry
☒	☐	MRI-based neuroimaging

Antibodies

Antibodies used

cFOS (Abcam 190289), CHAT (Proteintech 20747-1-AP), GABA (Sigma, 2025), GFAP (Abcam ab4674), HuC/D (Invitrogen a21271), NOS1 (SantaCruz Biotechnology sc-5302), NOS1 (Invitrogen 61-7000), PDGFRA (Cell Signaling 5241), PDGFRB (eBioscience 14-1402-82), S100B (Thermo Scientific RB 9018-P0), Serotonin (Sigma, S5545), STEM121 (Takara Bio, Y40410), TUBB3 (Millipore Sigma ab9354), TUBB3 (Biolegend 801202). nti chicken IgY (H+L) Alexa Fluor 488 (Invitrogen, A78948)
anti chicken IgY (H+L) Alexa Fluor 647 (Invitrogen, A78952)
anti mouse IgY (H+L) Alexa Fluor 488 (Invitrogen, A21202)
anti mouse IgY (H+L) Alexa Fluor 568 (Invitrogen, A10037)
anti mouse IgY (H+L) Alexa Fluor 647 (Invitrogen, A31571)
anti mouse IgG1a Alexa Fluor 568 (Invitrogen, A21124)
anti mouse IgG1a Alexa Fluor 647 (Invitrogen, A21240)
anti mouse IgG2a Alexa Fluor 488 (Invitrogen, A21131)
anti mouse IgG2b Alexa Fluor 568 (Invitrogen, A21144)
anti rabbit IgY (H+L) Alexa Fluor 647 (Invitrogen, A31573)
anti rat IgY (H+L) Alexa Fluor 647 (abcam, ab150155)

Validation

All primary and secondary antibodies were purchased from reputable manufacturers, previously validated by the manufacturers and cited in scientific literature. Antibody validations are performed by the vendors. Please find below information provided by the manufacturers.
"Anti-c-Fos antibody - BSA free is a rabbit polyclonal antibody used for detecting c-Fos in western blotting, ICC/IF, IHC-P, and IHC-FrFl. Suitable for human, mouse, and rat samples.- Cited in over 215 publications "
CHAT antibody: Proteintech: “20747-1-AP targets CHAT in WB, IHC, IF-P, IP, ELISA applications and shows reactivity with human, mouse, rat samples.Tested Reactivity: Human, Mouse, Rat, Cited Reactivity:Human, Mouse, Rat”
GABA antibody: Sigma, “Expression of GABA in neucortical cells harvested from the brains of E19 day old rat embyros was detected by immunofluorescence using rabbit anti-GABA antibody. Triple IF staining was performed with the anti-GABA antibody and two anti-GAD antibodies. Expression of GABA was analyzed in cells isolated from the pallium of various animals including rats, mice, rabbits, guinea pigs, and lizards by immunohistochemistry. IHC was performed using rabbit anti-GABA antibody at 1:1000 diluted in a solution of 0.01M PBS ph 7.4 + 0.5% triton-x100.
Rabbit anti-GABA antibody has been used for electron microscopy analysis in rat hippocampal tissues at a dilution of 1:4000. The antibody has also been used for immunocytochemistry applications at dilutions ranging from 1:100-1:750 in Drosophila and Periplaneta americana brain cells.”
GFAP antibody: Abcam: Anti-GFAP antibody is a chicken polyclonal antibody designed to detect glial fibrillary acidic protein (GFAP). Validated for ICC/IF, western blotting , IHC-P, and IHC-Fr applications. Suitable for mouse and rat samples. Cited in over 630 publications. Trusted by researchers since 2003”
HuC/D antibody, Invitrogen “Species reactivity: Avian, Chicken, Human, Zebrafish. A-21271 was successfully used to detect HuC/HuD in neurons differentiated from H9 ESC derived NSCs.”
NOS1 anitbody, SantaCruz “NOS1 Antibody (A-11) is a mouse monoclonal IgG1 κ NOS1 antibody, cited in 195 publications,raised against amino acids 2-300 of NOS1 of human origin, NOS1 Antibody (A-11) is recommended for detection of NOS1 of mouse, rat and human origin by WB, IP, IF and ELISA”
NOS1 antibody, Invitrogen “This antibody reacts with the ~160 kDa nNOS protein and does not exhibit any cross-reactivity with the related eNOS or iNOS proteins. During development reactivity was confirmed with a ~160 kD band on western blots of rat and mouse brain tissue lysates (20 µg). Species Reactivity:Mouse, Rat
Published species:Human, Mouse, Rat”
PDGFRA antibody, Cell Signaling “PDGF Receptor α (D13C6) XP® Rabbit mAb detects endogenous levels of PDGF receptor α protein. 73 citations. Species Reactivity: Human”
PDGFRB antibody, eBioscience “Applications Reported: The APB5 antibody has been reported for use in flow cytometric analysis and immunohistochemical staining of frozen tissue sections. It has also been reported in blocking of ligand binding. 87 published figures, Species Reactivity: Mouse, Published species:Fish, Human, Mouse”
PMP22 antibody, Abcam “Rabbit Polyclonal PMP22 antibody. Suitable for IHC-P and reacts with Rat samples. Immunogen corresponding to Synthetic Peptide within Human PMP22 aa 100-150 conjugated to Keyhole Limpet Haemocyanin.”
S100, Thermo Scientific “This antibody is excellent for staining of formalin-fixed, paraffin-embedded tissues. Species Reactivity: Human. Others-not tested. Immunogen: Recombinant protein encoding human S100 protein. Positive Control: Melanoma or Schwannoma”
Serotonin antibody, Sigma “Specificity
The antibody reacts with serotonin-containing fibers in paraformaldehyde perfusion-fixed, frozen sections of rat brain. The product specifically stains enterochromaffin cells in formalin-fixed, paraffin embedded sections of normal human appendix and serotonin-containing carcinoid tumors.”
STEM121 antibody, Takara Bio “STEM121 has been extensively used to detect the engraftment, migration and differentiation of human cells transplanted into mice and rats. The antibody can be used to quantify the location and number of engrafted cells and

the morphology of engrafted cells can be determined by immunohistochemistry using STEM121"
TUBB3 antibody, Abcam "species reactivity:rat, human, mouse, Specificity:Recognizes Beta III Tubulin Immunogen Epitope: (Similar to TuJ1 antigen), Synthetic peptides from human/rat Beta III Tubulin."
TUBB3 antibody, Biolegend "Verified Reactivity:Human, Mouse, Rat. This antibody was raised against microtubules derived from rat brain. 818 citations"

Eukaryotic cell lines

Policy information about cell lines and Sex and Gender in Research

Cell line source(s)	Human embryonic stem cell line H9 (female line, WiCell) WiCell: H9:hPSC Reg ID, WAe009-A, NIH Approval: NIHhESC-10-0062, Pub Med Abstract: Thomson, J. A. ``Embryonic Stem Cell Lines Derived from Human Blastocysts.`` Science 282.5391 (1998): 1145-147., Human induced pluripotent stem cells WTC11<br>(Coriell Institute, RRID: CVCL_Y803)WTC11: Source(s): Allen Institute; PubMed=34536661; PubMed=36965406<br>STR profile Markers:<br>Amelogenin X (Allen Institute)<br>X,Y (PubMed=34536661; PubMed=36965406)<br>CSF1PO 10,12<br>D2S1338 17,18<br>D3S1358 15,16<br>D5S818 9,10 (Allen Institute)<br>10 (PubMed=34536661; PubMed=36965406)<br>D7S820 10,12<br>D8S1179 11,13<br>D13S317 8,11 (Allen Institute)<br>8,12 (PubMed=34536661; PubMed=36965406)<br>D16S539 9,10<br>D18S51 12,21<br>D19S433 15,16<br>D21S11 29<br>FGA 22,25<br>Penta D 9,11<br>Penta E 13,15<br>TH01 6,7<br>TPOX 8,11<br>vWA 17,19
Authentication	Cell lines were obtained from reputable vendors and were previously validated by the vendors and cited.
Mycoplasma contamination	All cells were routinely checked and were negative for mycoplasma.
Commonly misidentified lines<br>(See ICLAC register)	No commonly misidentified cell lines were used in the study.

Animals and other research organisms

Policy information about studies involving animals; ARRIVE guidelines recommended for reporting animal research, and Sex and Gender in Research

Laboratory animals	Mus musculus, mice of both sexes, aged 4-8 weeks. Specified pathogen free (SPF) homozygote neuronal nitric oxide synthase knockout mice (B6.129S4-Nos1tm1Plh/J; nNos1-/-). Wild type male C57BL6 mice were obtained from Jackson Laboratory (RRID: IMSR_JAX:000664)
Wild animals	No wild animals were used in the study.
Reporting on sex	For transplantation studies, 14 of 31 mice were female and the rest were male, the animals were blindly assigned to experimental groups aiming for 1:1 sex in each group)
Field-collected samples	No field collected samples were used in the study.
Ethics oversight	Animals used for these studies were maintained, and the experiments performed, in accordance with the UK Animals (Scientific Procedures) Act 1986 and approved by the University College London Biological Services Ethical Review Process. Animal husbandry at UCL Biological Services was in accordance with the UK Home Office Certificate of Designation. All procedures followed the National Institutes of Health Guidelines for the Care and Use of Laboratory Animals and were approved by the Stanford University Administrative Panel on Laboratory Animal Care.

Note that full information on the approval of the study protocol must also be provided in the manuscript.

Plants

Seed stocks	N/A
Novel plant genotypes	N/A
Authentication	N/A

Flow Cytometry

Plots

Confirm that:

☒ The axis labels state the marker and fluorochrome used (e.g. CD4-FITC).

☒ The axis scales are clearly visible. Include numbers along axes only for bottom left plot of group (a 'group' is an analysis of identical markers).

☒ All plots are contour plots with outliers or pseudocolor plots.

☒ A numerical value for number of cells or percentage (with statistics) is provided.

Methodology

Sample preparation	For preparation of samples for flow cytometry analysis, cells were initially dissociated into single cell suspensions by accutase treatment (Stemcell Technologies, 07920, 30-60 min, 37 °C, 5% CO2) and then fixed and permeabilized using fixation/ permeabilization buffers (Foxp3/Transcription Factor Staining Buffer Set, 00-5523). Cells were stained with primary and secondary antibodies as described above for immunofluorescence.
Instrument	BD LSRFortessa cell analyzer
Software	Flowjo™ (FlowJo™ Software Version 8.7)
Cell population abundance	After rough filtering the cells based on the expected SSC and FSC distribution, all the used gates are shown in the manuscript.
Gating strategy	Gating strategy and assignment of positive and negative populations are shown throughout the presented data in the figures.

☒ Tick this box to confirm that a figure exemplifying the gating strategy is provided in the Supplementary Information.
